# Molecular and Serological Survey of Selected Viruses in Free-Ranging Wild Ruminants in Iran

**DOI:** 10.1371/journal.pone.0168756

**Published:** 2016-12-20

**Authors:** Farhid Hemmatzadeh, Wayne Boardman, Arezo Alinejad, Azar Hematzade, Majid Kharazian Moghadam

**Affiliations:** 1 School of Animal and Veterinary Sciences, The University of Adelaide, Adelaide, Australia; 2 School of Pharmacy and Medical Sciences, University of South Australia, Adelaide, Australia; 3 DVM graduate, Faculty of Veterinary Medicine, The University of Tehran, Tehran, Iran; 4 Faculty of Agriculture, Islamic Azad University, Shahrekord branch, Shahrekord, Iran; 5 Iran Department of Environment, Tehran, Iran; Katholieke Universiteit Leuven Rega Institute for Medical Research, BELGIUM

## Abstract

A molecular and serological survey of selected viruses in free-ranging wild ruminants was conducted in 13 different districts in Iran. Samples were collected from 64 small wild ruminants belonging to four different species including 25 Mouflon (*Ovis orientalis*), 22 wild goat (*Capra aegagrus*), nine Indian gazelle (*Gazella bennettii*) and eight Goitered gazelle (*Gazella subgutturosa*) during the national survey for wildlife diseases in Iran. Serum samples were evaluated using serologic antibody tests for Peste de petits ruminants virus (PPRV), Pestiviruses [Border Disease virus (BVD) and Bovine Viral Diarrhoea virus (BVDV)], Bluetongue virus (BTV), Bovine herpesvirus type 1 (BHV-1), and Parainfluenza type 3 (PI3). Sera were also ELISA tested for Pestivirus antigen. Tissue samples including spleen, liver, lung, tonsils, mesenteric and mediastinal lymph nodes and white blood cells (WBCs) were tested using polymerase chain reaction (PCR) for PPRV, Foot and Mouth Disease virus (FMDV), Pestivirus, BTV, Ovine herpesvirus type 2 (OvHV-2) and BHV-1. Serologic tests were positive for antibodies against PPRV (17%), Pestiviruses (2%) and BTV (2%). No antibodies were detected for BHV-1 or PI3, and no Pestivirus antigen was detected. PCR results were positive for PPRV (7.8%), FMDV (11%), BTV (3%), OvHV-2 (31%) and BHV-1 (1.5%). None of the samples were positive for Pestiviruses.

## Introduction

Habitat fragmentation, hunting, and infectious diseases often threaten biodiversity and may contribute to significant declines in wildlife populations [1, 2]. These populations can also act as reservoirs of transmissible viruses leading to ‘spill-over’ infections in livestock [3, 4]. In turn ‘spill-back’ infections from livestock can lead to disease outbreaks in wildlife [5]. To understand the role wildlife play in spill-over and spill-back events, veterinarians need to consider the ecological and epidemiological aspects of infectious disease agents [6, 7]. Although advanced in other countries, there has been limited monitoring of infectious disease agents in Iranian wildlife [8].

Wide climatic variations and untouched landscapes in Iran support a diverse mammalian fauna including 191 species from 93 genera and ten orders [9]. A number of ruminants inhabit different geographic regions, ranging from mountains with high annual rainfall to hot and dry deserts at low altitudes [10, 11]. Wild ruminants play a significant role in the ecology of transboundary disease in the world. Wild ruminants and livestock and sometimes humans share many similar pathogens [7]. Understanding the ecology of wildlife pathogens is critical to safety of livestock, humans and wildlife [12].

Most of the wild ruminants in Iran are on the International Union for the Conservation of Nature (IUCN) Red List as a direct result of infectious diseases, illegal hunting and drought [13].

Goitered gazelle, listed by the International Union for Conservation of Nature (IUCN) red list as vulnerable, are found in the Zargros region of Iran. Indian gazelle, protected in Iran but a considerable population of Indian gazelle are found in the Kavir National Park [13]. Both Wild Goat and Mouflon are listed by the IUCN red list as vulnerable and protected in Iran [14, 15]. Mouflon are distributed in mountainous parks and refuges including Kavir National Park, but there have been no recent population estimates. Wild goats, widely distributed throughout Iran live in mountainous areas, deserts and forested areas with estimates for Golestan National Park of only 2,500 animals [11, 14].

The majority of pathogens that cause disease outbreaks in wild ruminants worldwide are viral [6]. Peste des petits ruminants virus (PPRV), a *Morbillivirus* (family *Paramyxoviridae*) closely related to the eradicated rinderpest virus, causes the disease Peste des petits ruminants (PPR) which is considered to be one of the most significant livestock diseases in the Middle East, Africa and Asia [16, 17]. PPRV continues to spread across regions previously not affected [18]. Multiple outbreaks of PPR have occurred over the last ten years in Iran and neighbouring countries with devastating effects on the population of wild goats and sheep [19, 20]. PPR virus (PPRV) has also caused multiple deadly outbreaks in domestic small ruminants especially in western and north-central parts of Iran with significant economic losses [21, 22].

Foot and Mouth Disease (FMD) is caused by a single-stranded negative sense RNA virus that belongs to the genus Aphthovirus from the Picornaviridae family. The FMD virus (FMDV) contains seven different serotypes, O, A, C, Asia 1, Southern African Territories (SAT) 1, SAT 2 and SAT 3 [23]. Molecular and serological surveys in livestock have shown that FMDV has been endemic in Iran for more than 60 years [24]. In the last 20 years the most frequent FMDV isolate has been serotype A but serotype O and Asia 1 have been reported from major outbreaks in Iran and neighbouring countries [25, 26]. Serotypes O and A were responsible for recent outbreaks in 2014 and 2015 [27, 28]. Limited knowledge exists however, regarding the clinical signs, susceptibility and persistence of FMDV in wild ruminant populations in Iran. The existence of FMDV in wildlife has been reported in other countries. [29–31]. A clear variation of clinical features has been demonstrated in susceptible animals according to the serotype in natural and experimental infections [32, 33]. The role of wild animals in maintaining or circulating FMDV has been shown in different studies, but the presence of FMDV in Iranian wildlife has not been well demonstrated [31, 32, 34, 35].

Pestivirus infections in animals are caused by a group of viruses from the Flaviviridae family, genus pestivirus. Three different pestiviruses have been described, namely, bovine viral diarrhoea virus (BVDV), Border disease virus (BDV) and Classical Swine Fever Virus (CSFV) [36]. BVDV and BDV are widespread throughout the world and sero-epidemiological surveys of both these viruses in Iran showed widespread infection in cattle, sheep and goat populations [37, 38]. Possible transmission of pestiviruses between domestic animals and wild ruminants is considered a risk where they share pastures. [8, 36, 39]

Another important infection seen in domestic and wild ruminants is Bluetongue virus (BTV) an *Orbivirus* from the family *Reoviridae*. There are 24 antigenically distinct serotypes in the genus and differing levels of cross reactivity have been observed between different serotypes[40]. Epidemiological studies have demonstrated the importance of wild ruminants in the maintenance and circulation of BTV in both domestic and wild ruminants [41]. Sero-epidemiological investigations showed BTV is widespread in livestock in Iran [42–44]. However, no information is available on BTV in wild ruminants in Iran. Clinical and serological surveys in European wild ungulates have shown BTV infection does not cause clinical disease but they can act as reservoirs of the virus for other ruminant hosts [41]. BTV circulation between wild and domestic populations relies upon arthropod vectors such as *Culicoides* midges [45, 46] with disease circulating in both populations. [47].

Malignant Catarrhal Fever (MCF) is caused by members of subfamily Gammaherpesvirinae, genus Macavirus [48]. Alcelaphine herpesvirus 1 (AHV-1), causes a serious clinical form of the disease in Africa and in zoological parks where susceptible hosts are in contact with wildebeest [49]. Circulation of AHV-1 in wildlife species in Africa has been reported frequently and the risk of inter-species transmission of AHV-1, especially between wildlife and domestic livestock, is well understood [48]. The clinical form of Malignant Catarrhal Fever (MCF) due to Ovine herpesvirus 2 (OvHV-2) has been frequently reported in cattle and sheep in Iran [50, 51]. Domestic sheep are considered the major reservoir of OvHV-2 infection worldwide.[49, 50, 52]. The role of sheep in the transmission of OvHV-2 to wildlife is not well demonstrated, but cases of MCF in wildlife have been associated with both Caprine herpesvirus 2 (CpHV-2) and OvHV-2 [48, 53–55]. There has not been any previous evidence of OvHV-2 existing in wild ruminants in Iran.

Infectious bovine rhinotracheitis virus (IBRV) and parainfluenza type 3 virus (PI3V) are the main viral respiratory diseases in livestock worldwide [56, 57].

Infectious bovine rhinotracheitis (IBRV), caused by Bovine herpesvirus 1 (BHV-1), is associated with several clinical features in cattle. BHV-1 can produce a lifelong latent infection with occasional reactivation of the virus followed by viral shedding for several days. A serological positive response in animals is evidence of a latent infection [58]. Parainfluenza type 3 virus (PI3V), a RNA virus belonging to the *Paramyxovirus* family, is one of the most common viral respiratory infections in cattle. Although PI3V can cause primary respiratory disease in cattle, it usually leads to the development of secondary bacterial infections in the respiratory tract of large ruminants [59]. Both serological and PCR studies showed a high prevalence of these infections in livestock in Iran [60].The extent of these infections in wild ruminants is not well documented [1, 56].

The aim of this study was to investigate if there is evidence of these commonly seen livestock viruses in wild ruminants in the national parks and protected regions of Iran using serological and molecular techniques.

## Material and Methods

### Animals

The study animals comprised wild ruminants from four different species including 25 Mouflon (*Ovis orientalis*), 22 wild goat (*Capra aegagrus*), nine Indian gazelle (*Gazella bennettii*) and eight Goitered gazelle (*Gazella subgutturosa*).

Samples were collected from different districts in Iran; see Table 1. Animals were shot by authorized hunters during the national survey for wildlife diseases. All animals were killed and sampled under the national survey for wildlife disease programme which was funded and overseen by the Iran Department of Environment (DOE). All licences for hunting, necropsying, sampling and further analysis were issued by the Iran DOE.

**Table 1:** Regions of Iran and their geographic information system coordinates for all animals in the study.

		Species (the Scientific Names) and Number tested			
		Mouflon	Wild goat	Indian gazelle	Goitered gazelle
District	Geographic information	*(Ovis orientalis)*	*(Capra aegagrus)*	*(Gazella bennettii)*	*(Gazella subgutturosa)*
Golestan	37°31’N—53°04’E and 37.17°N—55.43°E	3	3	2	1
Kavir	51°25’N—53°3’E and 34°17’N—53°11’E	2	3	2	1
Tandooreh	37°15’N—37°35’E and 58°23’N—58°48’E	2	2	0	1
Khabr	28°59’N—28°25’E and 56°02’N—56°39’E	2	2	1	0
Bamo	29°53’N—29°36’E and 52°54’N—52°29’E	2	2	1	0
Khojir	35°45’N—35°36’E and 51°40’N—51°49’E	2	1	1	2
Mojen	36°28'37"N and 54°32'43"E	2	0	0	0
Shahzand	33°45'44"N and 49°43'21"E	1	1	0	0
Khomein	33°24’N—33°49’E and 49°38’N—49°49’E	2	1	0	0
Angooran	36°50’N—37°20’E and 74°15’N—74°50’E	2	2	0	0
Natanz	33°27' N and 81°48'E	1	1	0	0
Tooran	35°00’N—55°22’E and 35°22’N—57°02’E	2	2	1	2
Khosh Yeilagh	37°03' N and 55°54'E	2	2	1	1
Total		25	22	9	8

(http://www.unesco.org/mabdb/br/brdir/directory/contact.asp?code=IRA)

Based on morphological characteristics for age determination (teeth eruption method) [61] each individual animal was classified into three age groups: Age group 1; less than 1 year old, Age group 2; from 1 year to less than 2 years old and Age group 3; 2 years old and older.

### Samples

Whole blood was collected from the jugular vein, by cardiac aspiration or from the thoracic or abdominal cavities during fresh field necropsies by an authorized veterinarian from the Iran DOE. The collected blood was divided into two tubes, with and without anti-coagulant (EDTA) and labelled. Samples were transported, refrigerated, to the Virology laboratory at the Faculty of Veterinary Medicine at the University of Tehran within 24 hours.

The names of the regions and their geographic information system coordinates for all sampled animals and the age and sex of wildlife species tested in this study are shown in Tables 1 and 2.

**Table 2:** Species and age groups of animals examined in the study.

Species	Age categories			
	< 1 year	1 to < 2 years	≥ 2 years	Total
Mouflon *(Ovis orientalis)*	1	7	17	25
Wild goat *(Capra aegagrus)*	1	5	16	22
Indian gazelle *(Gazella bennettii)*	0	1	8	9
Goitered gazelle *(Gazella subgutturosa)*	0	0	8	8
Total	2	13	49	64

At the laboratory, serum was separated by centrifugation at 2000 RPM for 10 min. White blood cells from each blood sample was separated for antigen detection and RNA/ DNA isolation for molecular detection.

WBCs were isolated using Ficoll-Hypaque density gradient separation method. In brief, five mL of blood was diluted two-fold in sterile phosphate buffered saline (PBS) then added into the 10 mL of Ficoll-Hypaque (Sigma Aldrich), centrifuged for 400 × g at room temperature for 30 min. The cells were isolated and washed twice with PBS [62]. Aliquots of sera and WBCs were stored at −80°C until further analysis. Additional necropsies were performed at the mobile facilities in the field or at the diagnostic Virology laboratory at the Faculty of Veterinary Medicine at the University of Tehran. Spleen, liver, lung, tonsils and mesenchymal and mediastinal lymph nodes were collected for virus detection and stored on ice for transportation and at −80°C for long term storage.

### Ethics Statement

The study was approved by the Research and Ethics Committee of Iran Department of Environment. Animals were shot by authorized hunters and autopsied by veterinarians who specialized in wildlife studies during the national programme for survey of wildlife diseases. The hunting licences were issued by the Iran Department of Environment for the DOE qualified hunters.

### Serology and antigen detection

Serological tests used to detect antibodies included the following: a) PPR cELISA kit (Institute for Animal Health, Pirbright laboratory, UK), b) pestivirus blocking ELISA kit (BVDV) and (BDV) (Svanovir® BVDV p80-Ab, Boehringer Svanova Ingelheim, Uppsala, Sweden), c) Bluetongue competitive ELISA (ID VET, Montpellier, France) and d) Bovine Herpesvirus 1 (BHV-1) ELISA kit(Svanovir® IBR-Ab, Boehringer Svanova Ingelheim, Uppsala, Sweden) and e) virus neutralization test (VNT) for parainfluenza type 3 [60]. Pestivirus antigen detection kit, (Moredun Scientific Limited, Edinburgh, UK) was also used for detection of pestivirus antigens in buffy coat cells.

In all serological ELISA tests positive and negative control sera were included in each plate. In addition to the control samples (provided within the ELISA kits), two more known positive and negative samples were included in each round of ELISA or VNT tests. The positive and negative-control serum samples were serum from animals who had been tested at the diagnostic virology unit at the Razi Vaccine and Serum Research Institute or Virology laboratory at the Faculty of Veterinary Medicine at the University of Tehran, Iran and kept as standard control samples. Each of these serum samples were previously evaluated in different assays. For virus neutralization test, each serum sample was tested in two independent assay runs and serum samples with a titre of 1/64 or higher were considered positive [63]. All of the control samples were evaluated in each assay run and showed to be suitable for their intended purposes and served to monitor the assays consistency.

### Detection of viral nucleic acids

White blood cells and the fresh/frozen tissues from all animals were subject to DNA and RNA isolation. RNA extraction was performed using QIAamp Viral RNA Mini kit (QIAGEN, Hilden, Germany) according to the manufacturer's instructions. The cDNA was synthesized from extracted RNA using Moloney Murine Leukemia Virus-Reverse Transcriptase (MMLV-RT) and random primers (Invitrogen Corporation, San Diego, California, USA). Total DNA was isolated from the above mentioned samples using QIAamp DNA Mini Kit (QIAGEN, Hilden, Germany) according to the manufacturer's instructions. The synthesized cDNA and isolated DNA were saved as aliquots and stored at −80°C for further analysis.

Previously published PCR protocols were used to detect; a) PPR [64], b) FMD [27, 65], c) Pestivirus (BVDV and BDV) [66, 67], d) Bluetongue virus (BTV) [68, 69], e) Malignant Catarrhal Fever (MCF)[50] and f) Bovine Herpesvirus 1 (BHV-1) [70]. PCR products from all samples were analysed by electrophoresis in agarose gel and visualized under under ultra violet light. The selected PCR products from each assay from each animal were selected and purified using QIAquick Gel Extraction Kit (QIAGEN, Hilden, Germany) and submitted for sequencing for confirmation of the test results. The positive and negative controls used in this study were obtained from the following sources: positive BVDV control from Boehringer Svanova Ingelheim, Uppsala, Sweden, FMDV, PPRV and BHV-1 positive controls from Razi Vaccine and Serum Research Institute, Iran and OvHV-2, BDV and BTV positive controls from Faculty of Veterinary Medicine at the University of Tehran, Iran. Published primers on each were used for performing the molecular tests.

## Results

**Fig 1:**
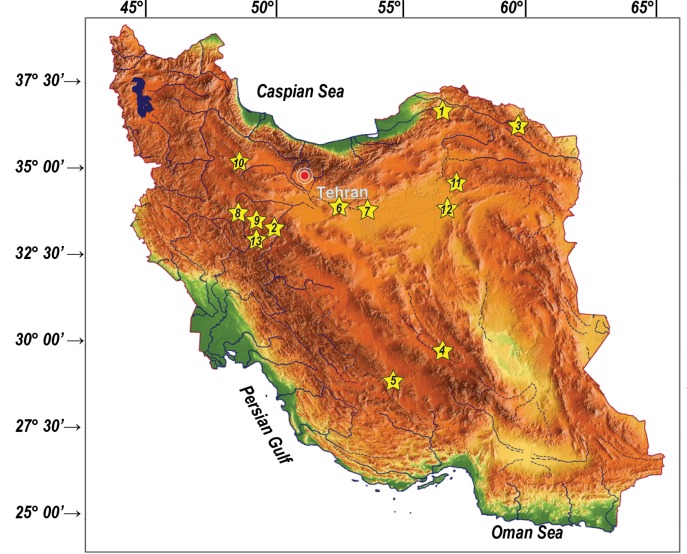
A map of Iran depicting sampling sites in the following protected regions: Golestan 1, Kavir 2, Tandooreh 3, Khabr 4, Bamo 5, Khojir 6 National Parks and Mujen 7, Shahzand 8, Khomein 9, Angooran 10, Tooran 11, Khosh Yeilagh 12 and Natanz 13. The source of the original map was from http://www.ginkgomaps.com.

Serological exposure of the 64 wild ruminants to PPR, BVD and BD, BTV, BHV-1, PI3 and pestivirus antigen is shown in Table 3 and PCR results for PPRV, FMDV, Pestivirus, BTV, OvHV-2 and BHV-1 are shown in Table 4. Seven out of 25 Mouflon (28%) and 4 out of 22 (18.1%) wild goats were positive for PPR virus antibodies. Four of seven PPR positive Mouflon were from Khojir (locality 6, Fig 1) and three from Bamo (locality 5, Fig 1) National Parks. Two of the pestivirus positive cases and one of the BTV positive cases were detected in Khojir and two other BTV cases were detected in Bamo National Park. An individual Mouflon in Bamo National Park was positive for both PPRV and BTV. Antibodies were not detected for other viruses. No positive results were found in Goitered gazelle and Indian gazelle.

**Table 3:** Results of serological antibody tests employed for the detection of PPRV, BVDV and BDV, BTV, BHV-1, PI3 and results of Pestivirus antigen with species and number of animals sampled.

	Positive viral serological test results					Positive Antigen test results
Species with total number of animals tested	PPRV (%)	Pestiviruses (%)	BTV (%)	BHV-1 (%)	PI3 (%)	Pestivirus (Antigen) (%)
Mouflon *(Ovis orientalis)* (25)	7 (28)	2 (8)	3 (12)	0 (0)	0 (0)	0 (0)
Wild goat *(Capra aegagrus)* (22)	4 (18)	0 (0)	0 (0)	0 (0)	0 (0)	0 (0)
Indian gazelle *(Gazella bennettii)* (9)	0 (0)	0 (0)	0 (0)	0 (0)	0 (0)	0 (0)
Goitered gazelle *(Gazella subgutturosa)* (8)	0 (0)	0 (0)	0 (0)	0 (0)	0 (0)	0 (0)
Total (64)	11 (17)	2 (3)	3 (4.5)	0 (0)	0 (0)	0 (0)

**Table 4:** Results of PCR tests employed for the detection of PPRV, FMDV, Pestivirus, BTV, OvHV-2 and BHV-1 with species and number of animals sampled in brackets.

	Positive PCR results					
Species with total number of animals tested	PPRV (%)	FMDV (%)	Pestivirus (%)	BTV (%)	OvHV-2 (%)	BHV-1 (%)
Mouflon *(Ovis orientalis)*(25)	4 (16)	4 (16)	0(0)	2 (8)	18 (40)	0(0)
Wild goat *(Capra aegagrus)* (22)	1 (5)	2 (10)	0(0)	0(0)	2(10)	0(0)
Indian gazelle *(Gazella bennettii)* (9)	0 (0)	1(11)	0(0)	0(0)	0(0)	1(11)
Goitered gazelle *(Gazella subgutturosa)*(8)	0(0)	0(0)	0(0)	0(0)	0(0)	0(0)
Total (64)	5 (7.8)	7 (11)	0 (0)	2 (3)	20 (31)	1 (1.5)

The PCR results for PPRV, FMDV, Pestivirus, BTV, OvHV-2 and BHV-1 are shown in Table 4. The most frequent viral infection detected in Mouflon and wild goats was OvHV-2. Seventy-six percent of tested WBC samples from Mouflon and 9% from wild goats were positive. The OvHV-2 infection was found in all of the 13 localities sampled. Four wild sheep and one wild goat were positive for PPRV by PCR in Khojir and Bamo National Parks. All of these five samples were positive by ELISA as well. Only one wild goat in Mujen protected region was found positive for PPRV by PCR. Two Mouflon from Bamo National Park were positive for BTV by PCR and one Indian gazelle was positive for BHV-1 virus in IBR specific PCR in WBCs [70].

Four mouflon, two wild goats and one Indian gazelle were positive for FMDV by PCR on tonsil samples. The only positive Indian gazelle for FMDV was from Tooran and the positive Mouflon were from Tooran (n = 1), Khojir (n = 2) and Angooran (n = 1). Two FMDV positive wild goats were from Khojir National park and Mujen protected region. All positive PCR tests for PPRV, FMDV, BHV-1 and four of the OvHV-2 samples were confirmed by sequencing. Edited nucleotide sequences were compared to the nucleotide sequence database for similarity using BLASTn search tool and was shown to have significant homology to the available sequences for the listed viruses. (S1–S5 Files).

## Discussion

This is the first survey of viral diseases in wild ruminants in Iran. It will provide valuable information for future research in disease ecology and risks associated with transmission of these viruses between wildlife and livestock.

Studies have shown that PPRV is endemic in Iran and the other neighbouring countries with multiple devastating outbreaks occurring in domestic and wild ruminants [16, 22]. More recently in 2015, the disease has been reported in range of domestic ruminants including goat, sheep and camels[71]. The antigenic similarity and cross protection of PPRV and rinderpest has been demonstrated in different studies [72]. Because of the disappearance of the cross-protective immunity caused by natural rinderpest infection, it has been suggested PPRV is now expanding into new regions and has the potential to cause severe epidemics in both domestic and wild small ruminant [73, 74]. As a result, expansion of PPR into wildlife populations could be the consequence of spread of the disease in livestock.[16] According to Iranian DOE, reports of a disease with considerable mortality in wild goats was observed in Khojir and Bamo National Parks in recent years [39]. Histopathological investigation at the time provided some evidence that the disease was PPR. In this study we confirmed serological and molecular diagnostic evidence of PPRV in both wild goats and Mouflon in these areas [21]. In both localities, no domestic livestock are allowed in the national parks, but wild goats and Mouflon are free to leave the park confines and graze adjacent to domestic animals. This information suggests a possible method of transmission for PPRV between livestock and wildlife. Interestingly, at the same time, the clinical form of PPR with high mortality has been reported in domestic sheep and goats in Tehran and Kerman provinces in areas close to Khojir and Bamo National parks [21, 22, 39], thus the likeliest source of infection for the wild ruminants in Khojir and Bamo National parks was from locally infected livestock or vice versa.

Border disease is considered as an endemic disease in domestic sheep and goats in Iran [38] and so Mouflon and wild goats could become infected through direct contact [75]. When planning to control livestock pestivirus infections managing existing pestivirus infections in wildlife also needs to be considered [76]. This study is the first report of Pestivirus seroprevalence in Mouflon in Iran. The necropsy of the two BDV seropositive animals did not reveal lesions suggestive of BD and PCR was negative; these results provide evidence of previous exposure to the virus but not active or persisting infection.

Foot and mouth disease has been reported in Mouflon, wild goats and Indian gazelles in several countries [31, 35]. As mentioned before, molecular surveys indicate the presence of O, A and Asia 1 FMD serotypes in livestock in Iran [27, 28]. In the present study, FMDV type A was detected by PCR test in the tonsils of Mouflon, wild goat and Indian gazelle, and PCR rusults were confirmed by gene sequencing. The positive test results in this study indicate that FMDV may remain as a persistent infection in wildlife. It has to be considered that FMDV can cause a persistent asymptomatic infection in ruminants with carriage lasting up to 3.5 years in cattle and 9 months in sheep [77]. Non vaccinated wild ruminants can act as a source of FMDV infection in livestock as well as wild ruminants [76]. Transmission of FMDV between wildlife and livestock, even in isolated areas, may be due to windborne infection or via fomites such as vehicles [77]. In this survey, all FMDV, PCR positive samples in wild ruminants were identical to the endemic Iranian type A isolate seen in domestic animals.[28].

Previous work in southeast Iran identified the serological existence of Bluetongue virus in domestic sheep with no reports of clinical signs [42, 44, 78]. Similarly, serological evidence of BTV was seen in wildlife without clinical signs in different countries of the world [42, 44, 78]. In both livestock and wildlife, BTV infection is characterized by various clinical forms from acute death, abortion, or no clinical signs [79]. In this study we did not find any link between the BTV infection and abortion or clinical disease.

In this survey all of the seropositive and PCR positive BT samples were seen in Mouflon from Bamo National Park. The locality of this survey overlaps geographically with the district where a high prevalence of BTV is seen in livestock [44].

There was no serological evidence for PI3 and IBR, the main viral infections of Iranian livestock, and there was just one Indian gazelle positive for BHV 1 using PCR. These viruses mainly cause disease in intensively managed livestock populations, so the lack of seroprevalence was expected in wildlife. In addition, BHV 1 infection is rarely seen in small ruminants and the clinical form is mainly associated with intensive feedlot or dairy production industries [80].

PCR tests showed a high prevalence of OvHV-2 in Mouflon. The virus does not produce clinical signs in small ruminants and there have been no reports of clinical disease in wild and domestic sheep and goats [49, 81]. In the non-African or sheep associated form of MCF caused by OvHV-2, small ruminants play a significant role as asymptomatic carriers of the virus which can be transmitted to cattle with serious consequences [49]. The OvHV-2 infection in sheep does not have any significant impact on wild sheep or goats but because of the sensitivity of the bovidae family to the virus, it may lead to considerable mortality in Indian and Goitered gazelles.[53, 81].

In conclusion, while there is clear evidence that transboundary disease viruses particularly FMDV, BTV and PPRV are circulating in wild ruminants. Further studies are required to investigate the transmission pathways and disease ecology between wildlife and livestock.

## Supporting Information

S1 File Supplementary file 1: Nucleotide alignment of partial VP7 gene of Bluetongue virus. GenBank accession numbers are shown at the left side of the figure and Iranian isolates are identified with double asterisk marks.
(PDF)

S2 File Supplementary file 2: Nucleotide alignment of partial Polyprotein gene of Foot-and-mouth disease virus A. GenBank accession numbers are shown at the left side of the figure and Iranian isolates are identified with double asterisk marks.
(PDF)

S3 File Supplementary file 3: Nucleotide alignment of partial major glycoprotein B (gB) gene of Bovine herpesvirus 1. GenBank accession numbers are shown at the left side of the figure and Iranian isolates are identified with double asterisk marks.
(PDF)

S4 File Supplementary file 4: Nucleotide alignment of partial tegument (teg) gene of Ovine herpesvirus 2. GenBank accession numbers are shown at the left side of the figure and Iranian isolates are identified with double asterisk marks.
(PDF)

S5 File Supplementary file 5: Nucleotide alignment of partial nucleoprotein (N) gene of Peste-des-petits-ruminants virus. GenBank accession numbers are shown at the left side of the figure and Iranian isolates are identified with double asterisk marks.
(PDF)
