# Supplementary material for: Molecular and Serological Survey of Selected Viruses in Free-Ranging Wild Ruminants in Iran
Source: PLoS One. 2016 Dec 20;11(12):e0168756. doi: 10.1371/journal.pone.0168756 (PMC5173247; doi:10.1371/journal.pone.0168756)
Supplement: S1 File — GenBank accession numbers are shown at the left side of the figure and Iranian isolates are identified with double asterisk marks. (PDF) [file pone.0168756.s001.pdf]

Supplementary file 1: Nucleotide alignment of partial VP7 gene of Bluetongue virus. GenBank accession numbers are shown at the left side of the figure and Iranian isolates are identified with double asterisk marks.

```
                                     10        20        30        40        50        60        70
                             ....|....|....|....|....|....|....|....|....|....|....|....|....|....|
gi|6176368|gb|AF188670.1|    GACCGATATCTCCAGATTATACCCAACATATGGCTACAATTGGTGTACTAGCGACGCCAGAGATACCTTT
gi|6176367|gb|AF188669.1|    GACCGATATCTCCAGATTATACCCAACATATGGCTACAATTGGTGTACTAGCGACGCCAGAGATACCTTT
gi|55420813|gb|AY776331.1|   GACCGATATCTCCAGATTATACCCAACATATGGCTACAATTGGTGTACTAGCGACGCCAGAGATACCTTT
gi|480327497|gb|JX272445.1|  GACCGATATCTCCAGATTATACCCAACATATGGCTACAATCGGCGTACTAGCGACGCCAGAGATACCTTT
gi|480327637|gb|JX272375.1|  GACCGATATCTCCAGATTATACCCAACATATGGCTACAATTGGTGTACTAGCGACGCCAGAGATACCCTT
gi|914341087|gb|KP821645.1|  GACCGATATCTCCAGATTATACCCAACATATGGCTACAATTGGTGTGCTAGCGACGCCAGAGATACCTTT
gi|914341085|gb|KP821644.1|  GACCGATATCTCCAGATTATACCCAACATATGGCTACAATTGGTGTGCTAGCGACGCCAGAGATACCTTT
gi|57013351|gb|AY841351.1|   GACCGATATCTCCAGATTATACCCAACATATGGCTACAATTGGTGTGCTAGCGACGCCAGAGATACCTTT
gi|914341107|gb|KP821655.1|  GACCGATATCTCCAGATTATACCCAACATATGGCTACAATTGGTGTGCTAGCGACGCCAGAGATACCTTT
gi|914341105|gb|KP821654.1|  GACCGATATCTCCAGATTATACCCAACATATGGCTACAATTGGTGTACTAGCAACGCCAGAGATACCCTT
gi|57013342|gb|AY839949.1|   GACCGATATCTCCAGATTATACCCAACATATGGCTACAATTGGTGTACTAGCAACGCCAGAGATACCCTT
gi|345846495|gb|JN255898.1|  GACCGATATCTCCAGATTATACCCAACATATGGCTACAATTGGTGTACTAGCAACGCCAGAGATACCCTT
gi|914341139|gb|KP821671.1|  GACCGATATCTCCAGATTATACCCAACATATGGCTACAATTGGTGTACTAGCAACGCCAGAGATACCCTT
gi|914341001|gb|KP821602.1|  GACCGATATCTCCAGATTATACCCAACATATGGCTACAATTGGTGTACTAGCAACGCCAGAGATACCTTT
gi|914341147|gb|KP821675.1|  GACCGATATCTCCAGATTATACCCAACATATGGCTACAATTGGTGTACTAGCGACGCCAGAGATACCTTT
gi|480327617|gb|JX272385.1|  GACCGATATCTCCAGATTATACCCAACATATGGCTACAATTGGTGTACTAGCGACGCCAGAGATACCTTT
**BTV.IR.2                   GACCGATATCTCCAGATTATACCCAACATATGGCTACAATTGGTGTACTAGCGACGCCAGAGATACCTTT
gi|224798943|gb|FJ745372.1|  GACCGATATCTCCAGATTATACCCAACATATGGCTACAATTGGTGTACTACCGACGCCACAGATACCTTT
****BTV.IR.1                 GACCGATATCTCCAGATTATACCCAACATATGGCTACAATTGGTGTACTACCGACGCCACAGATACCTTT

                                     80        90       100       110       120       130       140
                             ....|....|....|....|....|....|....|....|....|....|....|....|....|....|
gi|6176368|gb|AF188670.1|    TACAACGGAAGCGGCGAATGAGATTGCTCGCGTGACTGGGGAGACTTCGACGTGGGGACCAGCGCGTCAG
gi|6176367|gb|AF188669.1|    TACAACGGAAGCGGCGAATGAGATTGCTCGCGTGACTGAGGAGACTTCGACGTGGGGACCAGCGCGTCAG
gi|55420813|gb|AY776331.1|   TACAACGGAAGCGGCGAATGAGATTGCTCGCGTGACTGGGGAGACTTCGACGTGGGGACCAGCGCGTCAG
gi|480327497|gb|JX272445.1|  TACAACGGAAGCGGCGAATGAGATTGCTCGCGTGACTGGGGAGACTTCGACGTGGGGACCAGCGCGTCAG
gi|480327637|gb|JX272375.1|  TACAACGGAAGCGGCGAATGAGATTGCTCGCGTGACAGGGGAGACTTCGACGTGGGGACCAGCGCGCCAG
gi|914341087|gb|KP821645.1|  TACAACGGAAGCGGCGAATGAAATTGCTCGCGTGACTGGGGAGACTTCGACGTGGGGACCAGCGCGTCAG
gi|914341085|gb|KP821644.1|  TACAACGGAAGCGGCGAATGAAATTGCTCGCGTGACTGGGGAGACTTCGACGTGGGGACCAGCGCGTCAG
gi|57013351|gb|AY841351.1|   TACAACGGAAGCGGCGAATGAAATTGCTCGCGTGACTGGGGAGACTTCGACGTGGGGACCAGCGCGTCAG
gi|914341107|gb|KP821655.1|  TACAACGGAAGCGGCGAATGAAATTGCTCGCGTGACTGGGGAGACTTCGACGTGGGGACCAGCGCGTCAG
gi|914341105|gb|KP821654.1|  TACAACGGAAGCGGCGAACGAAATTGCTCGCGTGACTGGGGAGACTTCGACGTGGGGACCAGCACGTCAG
gi|57013342|gb|AY839949.1|   TACAACGGAAGCGGCGAACGAAATTGCTCGCGTGACTGGGGAGACTTCGACGTGGGGACCAGCACGTCAG
gi|345846495|gb|JN255898.1|  TACAACGGAAGCGGCGAACGAAATTGCTCGCGTGACTGGGGAGACTTCGACGTGGGGACCAGCACGTCAG
gi|914341139|gb|KP821671.1|  TACAACGGAAGCGGCGAACGAAATTGCTCGCGTGACTGGGGAGACTTCGACGTGGGGACCAGCACGTCAG
gi|914341001|gb|KP821602.1|  TACAACGGAAGCGGCAAACGAAATTGCTCGCGTGACTGGGGAGACTTCGACGTGGGGACCAGCACGTCAG
gi|914341147|gb|KP821675.1|  TACAACGGAAGCGGCAAACGAAATTGCTCGCGTGACTGGGGAGACTTCGACGTGGGGACCAGCGCGTCAG
gi|480327617|gb|JX272385.1|  TACAACGGAAGCGGCGAATGAAATTGCTCGCGTGACTGGGGAGACTTCGACGTGGGGACCGGCGCGTCAG
**BTV.IR.2                   TACAACGGAAGCGGCGAATGAAATTGCTCGCGTGACTGGGGAGACTTCGACGTGGGGACCGGCGCGTCAG
gi|224798943|gb|FJ745372.1|  TACAACGGAAGCGGCGAATGAAATTGCTCGCGTGACTGGGGAGACTTCGACGTGGGGACCGGCGCGTCAG
**BTV.IR.1                   TACAACGGAAGCGGCGAATGAAATTGCTCGCGTGACTGGGGAGACTTCGACGTGGGGACCGGCGCGTCAG

                                    150       160       170       180       190       200       210
                             ....|....|....|....|....|....|....|....|....|....|....|....|....|....|
gi|6176368|gb|AF188670.1|    CCCTATGGTTTTTTCCTTGAAACTGAAGAGACTTTCCACCCCGGGAGATGGTTCATGCGTGCCGCTCAAG
gi|6176367|gb|AF188669.1|    CCCTATGGTTTTTTCCTTGAAACTGAAGAGACTTTCCAACCCGGGAGATGGTTCATGCGTGCCGCTCAAG
gi|55420813|gb|AY776331.1|   CCCTATGGTTTTTTCCTTGAAACTGAAGAGACTTTCCAACCCGGGAGATGGTTCATGCGTGCCGCTCAAG
gi|480327497|gb|JX272445.1|  CCCTATGGTTTTTTCCTTGAAACTGAAGAGACTTTCCAACCCGGGAGATGGTTCATGCGTGCCGCTCAAG
gi|480327637|gb|JX272375.1|  CCCTATGGTTTTTTCCTTGAAACTGAAGAGACTTTCCAACCCGGGAGATGGTTCATGCGTGCCGCTCAAG
gi|914341087|gb|KP821645.1|  CCCTATGGTTTTTTCCTTGAAACTGAAGAGACTTTCCAACCTGGGAGATGGTTCATGCGCGCCGCTCAAG
gi|914341085|gb|KP821644.1|  CCCTATGGTTTTTTCCTTGAAACTGAAGAGACTTTCCAACCTGGGAGATGGTTCATGCGCGCCGCTCAAG
gi|57013351|gb|AY841351.1|   CCCTATGGTTTTTTCCTTGAAACTGAAGAGACTTTCCAACCTGGGAGATGGTTCATGCGCGCCGCTCAAG
gi|914341107|gb|KP821655.1|  CCCTATGGTTTTTTCCTTGAAACTGAAGAGACTTTCCAACCTGGGAGATGGTTCATGCGCGCCGCTCAAG
gi|914341105|gb|KP821654.1|  CCCTATGGTTTTTTCCTTGAAACTGAAGAGACTTTCCAACCTGGGAGATGGTTCATGCGTGCCGCTCAAG
gi|57013342|gb|AY839949.1|   CCCTATGGTTTTTTCCTTGAAACTGAAGAGACTTTCCAACCTGGGAGATGGTTCATGCGTGCCGCTCAAG
gi|345846495|gb|JN255898.1|  CCCTATGGTTTTTTCCTTGAAACTGAAGAGACTTTCCAACCTGGGAGATGGTTCATGCGTGCCGCTCAAG
gi|914341139|gb|KP821671.1|  CCCTATGGTTTTTTCCTTGAAACTGAAGAGACTTTCCAACCTGGGAGATGGTTCATGCGTGCCGCTCAAG
gi|914341001|gb|KP821602.1|  CCCTATGGTTTTTTCCTTGAAACTGAAGAGACTTTCCAACCTGGGAGATGGTTCATGCGTGCCGCTCAAG
gi|914341147|gb|KP821675.1|  CCCTATGGTTTTTTCCTTGAAACTGAAGAGACTTTCCAACCTGGGAGATGGTTCATGCGTGCCGCTCAAG
gi|480327617|gb|JX272385.1|  CCTTATGGTTTTTTCCTTGAAACTGAAGAGACTTACCAACCTGGGAGATGGTTCATGCGCGCCGCTCAAG
**BTV.IR.2                   CCTTATGGTTTTTTCCTTGAAACTGAAGAGACTTACCAACCTGGGAGATGGTTCATGCGCGCCGCTCAAG
gi|224798943|gb|FJ745372.1|  CCTTATGGTTTTTTCCTTGAAACTGAAGAGACTTACCAACCTGGGAGATGGTTCATGCGCGCCACTCAAG
**BTV.IR.1                   CCTTATGGTTTTTTCCTTGAAACTGAAGAGACTTACCAACCTGGGAGATGGTTCATGCGCGCCACTCAAG

                                    220       230       240       250       260       270       280
                             ....|....|....|....|....|....|....|....|....|....|....|....|....|....|
gi|6176368|gb|AF188670.1|    CAGTAACTGCGGTAGTGTGTGGTCCGGATATGATTCAAGTGTCACTGAATGCTGGAGCAAGAGGAGATGT
gi|6176367|gb|AF188669.1|    CAGTAACTGCGGTAGTGTGTGGTCCGGATATGATTCAAGTGTCACTGAATGCTGGAGCAAGAGGAGATGT
gi|55420813|gb|AY776331.1|   CAGTAACTGCGGTAGTGTGTGGTCCGGATATGATTCAAGTGTCACTGAATGCTGGAGCAAGAGGAGATGT
gi|480327497|gb|JX272445.1|  CAGTAACTGCGGTAGTGTGTGGTCCGGATATGATTCAAGTGTCACTGAATGCTGGAGCAAGAGGAGATGT
gi|480327637|gb|JX272375.1|  CAGTAACTGCGGTAGTGTGTGGTCCGGATATGATTCAAGTGTCACTGAATGCTGGAGCAAGAGGAGATGT
```

```
gi|914341087|gb|KP821645.1|  CAGTGACTGCGGTAGTGTGTGGTCCAGATATGATTCAAGTGTCACTGAATGCTGGAGCAAGAGGAGATGT
gi|914341085|gb|KP821644.1|  CAGTGACTGCGGTAGTGTGTGGTCCAGATATGATTCAAGTGTCACTGAATGCTGGAGCAAGAGGAGATGT
gi|57013351|gb|AY841351.1|   CAGTGACTGCGGTAGTGTGTGGTCCAGATATGATTCAAGTGTCACTGAATGCTGGAGCAAGAGGAGATGT
gi|914341107|gb|KP821655.1|  CAGTGACTGCGGTAGTGTGTGGTCCAGATATGATTCAAGTGTCACTGAATGCTGGAGCAAGAGGAGATGT
gi|914341105|gb|KP821654.1|  CAGTAACTGCGGTAGTGTGTGGTCCGGATATGATTCAAGTGTCGCTGAATGCTGGAGCGAGGGGAGATGT
gi|57013342|gb|AY839949.1|   CAGTAACTGCGGTAGTGTGTGGTCCGGATATGATTCAAGTGTCGCTGAATGCTGGAGCGAGGGGAGATGT
gi|345846495|gb|JN255898.1|  CAGTAACTGCGGTAGTGTGTGGTCCGGATATGATTCAAGTGTCGCTGAATGCTGGAGCGAGGGGAGATGT
gi|914341139|gb|KP821671.1|  CAGTAACTGCGGTAGTGTGTGGTCCGGATATGATTCAAGTGTCGCTGAATGCTGGAGCGAGGGGAGATGT
gi|914341001|gb|KP821602.1|  CAGTAACTGCGGTAGTGTGTGGTCCGGATATGATTCAAGTGTCGCTGAATGCTGGAGCGAGAGGAGATGT
gi|914341147|gb|KP821675.1|  CAGTAACTGCGGTAGTGTGTGGTCCAGATATGATTCAAGTGTCGCTGAATGCTGGAGCGAGAGGAGATGT
gi|480327617|gb|JX272385.1|  CAGTAACTGCGGTAGTGTGTGGTCCGGATATGATTCAAGTGTCACTAAATGCTGGAGCAAGAGGAGATGT
**BTV.IR.2                     CAGTAACTGCGGTAGTGTGTGGTCCGGATATGATTCAAGTGTCACTAAATGCTGGAGCAAGAGGAGATGT
gi|224798943|gb|FJ745372.1|  CAGAAACTGAGGTAGTGTGAGGTCCGGATATGATTCAAGTGTCAATAAATGCTGGAGCAAGAGGAAATGT
**BTV.IR.1                     CAGAAACTGAGGTAGTGTGAGGTCCGGATATGATTCAAGTGTCAATAAATGCTGGAGCAAGAGGAAATGT

                                     290       300       310       320       330       340       350
                             ....|....|....|....|....|....|....|....|....|....|....|....|....|....|
gi|6176368|gb|AF188670.1|    GCAGCAGATATTTCAGGGTCGTAACGACCCCATGATGATATATCTAGTTTGGAGAAGAATTGAAAACTTC
gi|6176367|gb|AF188669.1|    GCAGCAGATATTTCAGGGTCGTAACGACCCCATGATGATATATCTAGTTTGGAGAAGAATTGAAAACTTC
gi|55420813|gb|AY776331.1|   GCAGCAGATATTTCAGGGTCGTAACGACCCCATGATGATATATCTAGTTTGGAGAAGAATTGAAAACTTC
gi|480327497|gb|JX272445.1|  GCAGCAGATATTTCAGGGTCGTAACGACCCCATGATGATATATCTAGTTTGGAGAAGAATTGAAAACTTC
gi|480327637|gb|JX272375.1|  GCAGCAGATATTTCAGGGTCGTAACGACCCCATGATGATATATCTAGTTTGGAGAAGAATTGAAAACTTC
gi|914341087|gb|KP821645.1|  GCAGCAGATATTTCAGGGTCGTAATGATCCCATGATGATATATCTAGTTTGGAGGAGAATTGAAAACTTC
gi|914341085|gb|KP821644.1|  GCAGCARATATTTCAGGGTCGTAATGATCCCATGATGATATATCTAGTTTGGAGGAGAATTGAAAACTTC
gi|57013351|gb|AY841351.1|   GCAGCAGATATTTCAGGGTCGTAATGATCCCATGATGATATATCTAGTTTGGAGGAGAATTGAAAACTTC
gi|914341107|gb|KP821655.1|  GCAGCAGATATTTCAGGGTCGTAATGATCCCATGATGATATATCTAGTTTGGAGGAGAATTGAAAACTTC
gi|914341105|gb|KP821654.1|  GCAGCAGATATTTCAAGGTCGTAATGATCCCATGATGATATATCTAGTTTGGAGAAGAATTGAAAACTTC
gi|57013342|gb|AY839949.1|   GCAGCAGATATTTCAAGGTCGTAATGATCCCATGATGATATATCTAGTTTGGAGAAGAATTGAAAACTTC
gi|345846495|gb|JN255898.1|  GCAGCAGATATTTCAAGGTCGTAATGATCCCATGATGATATATCTAGTTTGGAGAAGAATTGAAAACTTC
gi|914341139|gb|KP821671.1|  GCAGCAGATATTTCAAGGTCGTAATGATCCCATGATGATATATCTAGTTTGGAGAAGAATTGAAAACTTC
gi|914341001|gb|KP821602.1|  GCAGCAGATATTTCAAGGTCGTAATGATCCCATGATGATATATCTAGTTTGGAGAAGAATTGAAAACTTC
gi|914341147|gb|KP821675.1|  GCAGCAGATATTTCAGGGTCGTAATGATCCCATGATGATATATCTAGTTTGGAGAAGAATTGAAAACTTC
gi|480327617|gb|JX272385.1|  GCAGCAGATATTTCAGGGTCGTAATGATCCCATGATGATATATCTAGTTTGGAGAAGAATTGAAAACTTC
**BTV.IR.2                   GCAGCAGATATTTCAGGGTCGTAATGATCCCATGATGATATATCTAGTTTGGAGAAGAATTGAAAACTTC
gi|224798943|gb|FJ745372.1|  GCAGCAGATATTTCAGGGTCGTAATGATCCCATGATAATATATCTAGTTTGGAGAAGAATTGAAAACTTC
**BTV.IR.1                   GCAGCAGATATTTCAGGGTCGTAATGATCCCATGATAATATATCTAGTTTGGAGAAGAATTGAAAACTTC

                                     360       370       380       390       400       410       420
                             ....|....|....|....|....|....|....|....|....|....|....|....|....|....|
gi|6176368|gb|AF188670.1|    GCGATGGCGCAGGGTAACTCACAGCAAACTCAAGCAGGCGTGACTGTTAGCGTTGGTGGAGTAGATATGC
gi|6176367|gb|AF188669.1|    GCGATGGCGCAGGGTAACTCACAGCAAACTCAAGCAGGCGTGACTGTTAGCGTTGGTGGAGTAGATATGC
gi|55420813|gb|AY776331.1|   GCGATGGCGCAGGGTAACTCACAGCAAACTCAAGCAGGCGTGACTGTTAGCGTTGGTGGAGTAGATATGC
gi|480327497|gb|JX272445.1|  GCGATGGCGCAGGGTAACTCACAGCAAACTCAAGCAGGCGTGACTGTTAGCGTTGGTGGAGTAGATATGC
gi|480327637|gb|JX272375.1|  GCGATGGCGCAAGGTAACTCACAGCAAACTCAAGCAGGTGTGACTGTTAGCGTTGGCGGAGTAGATATGC
gi|914341087|gb|KP821645.1|  GCGATGGCGCAGGGTAACTCACAGCAAACTCAAGCAGGCGTGACTGTTAGCGTTGGCGGAGTAGACATGC
gi|914341085|gb|KP821644.1|  GCGATGGCGCAGGGTAACTCACAGCAAACTCAAGCAGGCGTGACTGTTAGCGTTGGCGGAGTAGACATGC
gi|57013351|gb|AY841351.1|   GCGATGGCGCAGGGTAACTCACAGCAAACTCAAGCAGGCGTGACTGTTAGCGTTGGCGGAGTAGACATGC
gi|914341107|gb|KP821655.1|  GCGATGGCGCAGGGTAACTCACAGCAAACTCAAGCAGGCGTGACTGTTAGCGTTGGCGGAGTAGACATGC
gi|914341105|gb|KP821654.1|  GCAATGGCGCAGGGTAACTCACAGCAAACTCAAGCAGGCGTGACTGTTAGCGTTGGCGGAGTAGACATGC
gi|57013342|gb|AY839949.1|   GCAATGGCGCAGGGTAACTCACAGCAAACTCAAGCAGGCGTGACTGTTAGCGTTGGCGGAGTAGACATGC
gi|345846495|gb|JN255898.1|  GCAATGGCGCAGGGTAACTCACAGCAAACTCAAGCAGGCGTGACTGTTAGCGTTGGCGGAGTAGACATGC
gi|914341139|gb|KP821671.1|  GCAATGGCGCAGGGTAACTCACAGCAAACTCAAGCAGGCGTGACTGTTAGCGTTGGCGGAGTAGACATGC
gi|914341001|gb|KP821602.1|  GCAATGGCGCAGGGTAACTCACAGCAGACTCAAGCAGGCGTGACTGTTAGCGTTGGCGGAGTAGACATGC
gi|914341147|gb|KP821675.1|  GCAATGGCGCAGGGTAACTCACAGCAAACTCAAGCAGGCGTGACTGTTAGCGTTGGCGGAGTAGACATGC
gi|480327617|gb|JX272385.1|  GCGATGGCGCAGGGTAACTCGCAGCAAACTCAAGCAGGCGTGACTGTTAGCGTTGGCGGAGTAGACATGC
**BTV.IR.2                   GCGATGGCGCAGGGTAACTCGCAGCAAACTCAAGCAGGCGTGACTGTTAGCGTTGGCGGAGTAGACATGC
gi|224798943|gb|FJ745372.1|  GCGATGGCGCAGGGTAACTCGCAGCAAACTCAAGCAGGTGTGACTGTTAGCGTTGGCGGAGTAGACATGC
**BTV.IR.1                   GCGATGGCGCAGGGTAACTCGCAGCAAACTCAAGCAGGTGTGACTGTTAGCGTTGGCGGAGTAGACATGC

                                     430       440       450       460       470       480       490
                             ....|....|....|....|....|....|....|....|....|....|....|....|....|....|
gi|6176368|gb|AF188670.1|    GGGCGGGGCGTATCATAGCGTGGGATGGACAGGCTGCTCTACATGTGCACAATCCAACACAACAGAATGC
gi|6176367|gb|AF188669.1|    GGGCGGGGCGTATCATAGCGTGGGATGGACAGGCTGCTCTACATGTGCACAATCCAACACAACAGAATGC
gi|55420813|gb|AY776331.1|   GGGCGGGGCGTATCATAGCGTGGGATGGACAGGCTGCTCTACATGTGCACAATCCAACACAACAGAATGC
gi|480327497|gb|JX272445.1|  GGGCGGGGCGTATCATAGCGTGGGATGGACAGGCTGCTCTACATGTGCACAATCCAACACAACAGAATGC
gi|480327637|gb|JX272375.1|  GGGCGGGGCGTATCATAGCGTGGGATGGACAGGCCGCTCTACATGTGCACAATCCAACACAACAGAATGC
gi|914341087|gb|KP821645.1|  GGGCGGGGCGTATCATAGCGTGGGATGGGCAAGCTGCTCTACATGTGCATAATCCCACACAACAGAATGC
gi|914341085|gb|KP821644.1|  GGGCGGGGCGTATCATAGCGTGGGATGGGCAAGCTGCTCTACATGTGCATAATCCCACACAACAGAATGC
gi|57013351|gb|AY841351.1|   GGGCGGGGCGTATCATAGCGTGGGATGGGCAAGCTGCTCTACATGTGCATAATCCCACACAACAGAATGC
gi|914341107|gb|KP821655.1|  GGGCGGGGCGTATCATAGCGTGGGATGGGCAAGCTGCTCTACATGTGCATAATCCCACACAACAGAATGC
gi|914341105|gb|KP821654.1|  GGGCAGGGCGTATCATAGCGTGGGATGGACAAGCTGCTCTTCATGTGCATAATCCAACACAACAGAATGC
gi|57013342|gb|AY839949.1|   GGGCAGGGCGTATCATAGCGTGGGATGGACAAGCTGCTCTTCATGTGCATAATCCAACACAACAGAATGC
gi|345846495|gb|JN255898.1|  GGGCAGGGCGTATCATAGCGTGGGATGGACAAGCTGCTCTTCATGTGCATAATCCAACACAACAGAATGC
gi|914341139|gb|KP821671.1|  GGGCAGGGCGTATCATAGCGTGGGATGGACAAGCTGCTCTTCATGTGCATAATCCAACACAACAGAATGC
gi|914341001|gb|KP821602.1|  GGGCAGGGCGTATCATAGCGTGGGATGGACAAGCTGCTCTACATGTGCATAATCCAACACAACAGAATGC
gi|914341147|gb|KP821675.1|  GGGCAGGGCGTATCATAGCGTGGGATGGACAAGCCGCTCTACATGTGCATAATCCAACACAACAGAATGC
gi|480327617|gb|JX272385.1|  GGGCGGGGCGTATTATAGCGTGGGACGGACAAGCAGCTCTACATGTGCATAATCCAACACAACAGAACGC
```

```
**BTV.IR.2                       GGGCGGGGCGTATTATAGCGTGGGACGGACAAGCAGCTCTACATGTGCATAATCCAACACAACAGAACGC
gi|224798943|gb|FJ745372.1|      GGGCGGGGCGTATTATAGCGTGGGACGGACAAGCAGCTCTACATGTGCATAATCCAACACAACAGAACGA
**BTV.IR.1                       GGGCGGGGCGTATTATAGCGTGGGACGGACAAGCAGCTCTACATGTGCATAATCCAACACAACAGAACGA

                                          500       510       520       530       540       550       560
                                 ....|....|....|....|....|....|....|....|....|....|....|....|....|....|
gi|6176368|gb|AF188670.1|        GATGGTTCAGATACAAGTCGTGTTCTACATTTCTATGGATAAGACCTTAAATCAATACCCTGCCTTGACT
gi|6176367|gb|AF188669.1|        GATGGTTCAGATACAAGTCGTGTTCTACATTTCTATGGATAAGACCTTAAATCAATACCCTGCCTTGACT
gi|55420813|gb|AY776331.1|       GATGGTTCAGATACAAGTCGTGTTCTACATTTCTATGGATAAGACCTTAAATCAATACCCTGCCTTGACT
gi|480327497|gb|JX272445.1|      GATGGTTCAGATACAAGTTGTGTTCTACATTTCTATGGATAAGACCTTAAATCAATACCCTGCCTTGACT
gi|480327637|gb|JX272375.1|      GATGGTTCAGATACAAGTTGTGTTTTACATTTCTATGGATAAGACCTTAAATCAATATCCTGCCTTGACT
gi|914341087|gb|KP821645.1|      GATGGTCCAAATACAAGTTGTGTTTTATATTTCCATGGATAAGACCTTAAATCAATACCCTGCCTTGACT
gi|914341085|gb|KP821644.1|      GATGGTCCAAATACAAGTTGTGTTTTATATTTCCATGGATAAGACCTTAAATCAATACCCTGCCTTGACT
gi|57013351|gb|AY841351.1|       GATGGTCCAAATACAAGTTGTGTTTTATATTTCCATGGATAAGACCTTAAATCAATACCCTGCCTTGACT
gi|914341107|gb|KP821655.1|      GATGGTCCAAATACAAGTTGTGTTTTATATTTCCATGGATAAGACCTTAAATCAATACCCTGCCTTGACT
gi|914341105|gb|KP821654.1|      GATGGTCCAGATACAAGTTGTATTTTACATCTCCATGGATAAGACCTTAAATCAATACCCCGCCTTGACT
gi|57013342|gb|AY839949.1|       GATGGTCCAGATACAAGTTGTATTTTACATCTCCATGGATAAGACCTTAAATCAATACCCCGCCTTGACT
gi|345846495|gb|JN255898.1|      GATGGTCCAGATACAAGTTGTATTTTACATCTCCATGGATAAGACCTTAAATCAATACCCCGCCTTGACT
gi|914341139|gb|KP821671.1|      GATGGTCCAGATACAAGTTGTATTTTACATCTCCATGGATAAGACCTTAAATCAATACCCCGCCTTGACT
gi|914341001|gb|KP821602.1|      GATGGTCCAGATACAAGTTGTATTTTACATTTCCATGGATAAGACCTTAAATCAATACCCTGCCTTGACT
gi|914341147|gb|KP821675.1|      GATGGTCCAGATACAAGTTGTATTTTACATTTCCATGGATAAGACCTTAAATCAATACCCTGCCTTGACT
gi|480327617|gb|JX272385.1|      GATGGTCCAGATACAAGTTGTGTTTTACATTTCTATGGATAAGACCTTAAATCAATACCCTGCCTTGACT
**BTV.IR.2                       GATGGTCCAGATACAAGTTGTGTTTTACATTTCTATGGATAAGACCTTAAATCAATACCCTGCCTTGACT
gi|224798943|gb|FJ745372.1|      GAGGGTCCAGATACAAGTAGGGTATTACAATTCTATGGATAAGACCTAAAATCAATACCCGGCCTTGACT
**BTV.IR.1                       GAGGGTCCAGATACAAGTAGGGTATTACAATTCTATGGATAAGACCTAAAATCAATACCCGGCCTTGACT

                                          570       580       590       600       610       620
                                 ....|....|....|....|....|....|....|....|....|....|....|....|...
gi|6176368|gb|AF188670.1|        GCTGAAATCTTTAATGTTTATAGCTTCAGAGATCACACATGGCACGGGTTGAGGACGGCTATA
gi|6176367|gb|AF188669.1|        GCTGAAATCTTTAATGTTTATAGCTTCAGAGATCACACATGGCACGGGTTGAGGACGGCTATA
gi|55420813|gb|AY776331.1|       GCTGAAATCTTTAATGTTTATAGCTTCAGAGATCACACATGGCACGGGTTGAGGACGGCTATA
gi|480327497|gb|JX272445.1|      GCTGAAATCTTTAATGTTTATAGCTTCAGAGATCACACATGGCACGGGTTGAGGACGGCTATA
gi|480327637|gb|JX272375.1|      GCTGAAATCTTTAATGTTTATAGCTTCAGAGATCACACATGGCACGGGTTGAGGACGGCTATA
gi|914341087|gb|KP821645.1|      GCTGAAATCTTTAATGTTTATAGCTTCAGAGATCACACATGGCACGGGTTGAGGACGGCTATA
gi|914341085|gb|KP821644.1|      GCTGAAATCTTTAATGTTTATAGCTTCAGAGATCACACATGGCACGGGTTGAGGACGGCTATA
gi|57013351|gb|AY841351.1|       GCTGAAATCTTTAATGTTTATAGCTTCAGAGATCACACATGGCACGGGTTGAGGACGGCTATA
gi|914341107|gb|KP821655.1|      GCTGAAATCTTTAATGTTTATAGCTTCAGAGATCACACATGGCACGGGTTGAGGACGGCTATA
gi|914341105|gb|KP821654.1|      GCTGAAATCTTTAATGTTTATAGCTTCAGAGATCACACATGGCACGGGTTGAGGACGGCTATA
gi|57013342|gb|AY839949.1|       GCTGAAATCTTTAATGTTTATAGCTTCAGAGATCACACATGGCACGGGTTGAGGACGGCTATA
gi|345846495|gb|JN255898.1|      GCTGAAATCTTTAATGTTTATAGCTTCAGAGATCACACATGGCACGGGTTGAGGACGGCTATA
gi|914341139|gb|KP821671.1|      GCTGAAATCTTTAATGTTTATAGCTTCAGAGATCACACATGGCACGGGTTGAGGACGGCTATA
gi|914341001|gb|KP821602.1|      GCTGAAATCTTTAATGTTTATAGCTTCAGAGATCACACATGGCACGGGTTGAGGACGGCTATA
gi|914341147|gb|KP821675.1|      GCTGAAATCTTTAATGTTTATAGCTTCAGAGATCACACATGGCACGGGTTGAGGACGGCTATA
gi|480327617|gb|JX272385.1|      GCTGAAATCTTTAATGTTTATAGCTTCAGAGATCACACATGGCACGGGTTGAGGACGGCTATA
**BTV.IR.2                       GCTGAAATCTTTAATGTTTATAGCTTCAGAGATCACACATGGCACGGGTTGAGGACGGCTATA
gi|224798943|gb|FJ745372.1|      GCAGAAATCTTTAATGTTTATAGCTTCAGAGATCACACATGGCACGGGTTGAGGACGGCTATA
**BTV.IR.1                       GCAGAAATCTTTAATGTTTATAGCTTCAGAGATCACACATGGCACGGGTTGAGGACGGCTATA
```
