# Supplementary material for: Molecular and Serological Survey of Selected Viruses in Free-Ranging Wild Ruminants in Iran
Source: PLoS One. 2016 Dec 20;11(12):e0168756. doi: 10.1371/journal.pone.0168756 (PMC5173247; doi:10.1371/journal.pone.0168756)
Supplement: S2 File — GenBank accession numbers are shown at the left side of the figure and Iranian isolates are identified with double asterisk marks. (PDF) [file pone.0168756.s002.pdf]

Supplementary file 2: Nucleotide alignment of partial Polyprotein gene of Foot-and-mouth disease virus A. GenBank accession numbers are shown at the left side of the figure and Iranian isolates are identified with double asterisk marks.

```
                                      10        20        30        40        50        60        70
                               ....|....|....|....|....|....|....|....|....|....|....|....|....|....|
gi|1016883780|gb|KX002203.1|   TTCCTGCTTCTTTCAACTACGGTGCAATCAAGGCCGACAACGTGCACGAGCTTCTCGTGCGCATGAAACG
gi|1016883774|gb|KX002200.1|   TTCCTGCTTCTTTCAACTACGGTGCAATCAAGGCCGACAACGTGCACGAGCTTCTCGTGCGCATGAAACG
gi|1016883753|gb|KX002188.1|   TTCCTGCTTCTTTCAACTACGGTGCAATCAAGGCCGACAACGTGCACGAGCTTCTCGTGCGCATGAAACG
gi|1016883773|gb|KX002199.1|   TTCCTGCTTCTTTCAACTACGGTGCAATCAAGGCCGACAACGTGCACGAGCTTCTCGTGCGCATGAAACG
gi|1016883764|gb|KX002194.1|   TTCCTGCTTCTTTCAACTACGGTGCAATCAAGGCCGACAACGTGCACGAGCTTCTCGTGCGCATGAAACG
gi|1016883757|gb|KX002190.1|   TTCCTGCTTCTTTCAACTACGGTGCAATCAAGGCCGACAACGTGCACGAGCTTCTCGTGCGCATGAAACG
gi|1016883766|gb|KX002195.1|   TTCCTGCTTCTTTCAACTACGGTGCAATCAAGGCCGACAACGTGCACGAGCTTCTCGTGCGCATGAAACG
gi|1016883770|gb|KX002197.1|   TTCCTGCTTCTTTCAACTACGGTGCAATCAAGGCCGACAACGTGCACGAGCTCCTCGTGCGCATGAAACG
gi|1016883782|gb|KX002204.1|   TTCCTGCTTCTTTCAACTACGGTGCAATCAAGGCCGACAACGTGCACGAGCTTCTCGTGCGCATGAAACG
gi|1016883749|gb|KX002186.1|   TTCCTGCTTCTTTCAACTACGGTGCAATCAAGGCCGACAACGTGCACGAGCTTCTCGTGCGCATGAAACG
gi|1016883784|gb|KX002205.1|   TTCCTGCTTCTTTCAACTACGGTGCAATCAAGGCCGACAACGTGCACGAGCTTCTCGTGCGCATGAAACG
gi|1016883751|gb|KX002187.1|   TTCCTGCTTCTTTCAACTACGGTGCAATCAAGGCCGACAACGTGCACGAGCTTCTCGTGCGCATGAAACG
gi|1016883771|gb|KX002198.1|   TTCCTGCTTCTTTCAACTACGGTGCAATCAAGGCCGACAACGTGCACGAGCTTCTCGTGCGCATGAAACG
gi|1016883760|gb|KX002192.1|   TTCCTGCTTCTTTCAACTACGGTGCAATCAAGGCCGACAACGTGCACGAGCTTCTCGTGCGCATGAAACG
gi|1016883778|gb|KX002202.1|   TTCCTGCTTCTTTCAACTACGGTGCAATCAAGGCCGACAACGTGCACGAGCTTCTCGTGCGCATGAAACG
gi|1016883768|gb|KX002196.1|   TTCCTGCTTCTTTCAACTACGGTGCAATCAAGGCCGACAACGTGCACGAGCTTCTCGTGCGCATGAAACG
gi|1016883758|gb|KX002191.1|   TTCCTGCTTCTTTCAACTACGGTGCGGTCAAGGCCGACAACGTGCACGAGCTTCTCGTGCGCATGAAACG
**FMD1                         TCCCTGCCTCTTTCAACTTTGGTGCAATCCGGGCCACTAACATCAGTGAGCTTCTGGTGCGCATGAAGCG
gi|225032211|gb|FJ755018.1     TCCCTGCCTCTTTCAACTTTGGTGCAATCCGGGCCACTAACATCAGTGAGCTTCTGGTGCGCATGAAGCG
**FMD7                         TCCCTGCCTCTTTCAACTTTGGTGCAATCCGGGCCACTAACATCAGTGAGCTTCTGGTGCGCATGAAGCG
**FMD3                         TCCCTGCCTCTTTCAACTTTGGTGCAATCCAGGCCACTAACATCAGTGAGCTTCTGGTGCGCATGAAGCG
gi|225032219|gb|FJ755022.1     TCCCTGCCTCTTTCAACTTTGGTGCAATCCAGGCCACTAACATCAGTGAGCTTCTGGTGCGCATGAAGCG
gi|62083795|gb|AY960760.1|     TCCCTGCCTCTTTCAACTTTGGTGCAATCCAGGCCACTAACATCAGTGAGCTTCTGGTGCGCATGAAGCG
**FMD2                         TCCCTGCCTCTTTCAACTTTGGTGCAATCCAGGCCACTAACATCAGTGAGCTTCTGGTGCGCATGAAGCG
gi|126015403|gb|EF405982.1     TCCCTGCCTCTTTCAACTTTGGCGCAATCCGGGCCACTGACATCAGTGAGCTTCTTGTGCGCATGAAGCG
**FMD5                         TCCCTGCCTCTTTCAACTTTGGCGCAATCCGGGCCACTGACATCAGTGAGCTTCTTGTGCGCATGAAGCG
gi|46810838|gb|AY593791.1|     TCCCTGCCTCTTTCAACTTTGGCGCAATCCGGGCCACTGACATCAGTGAGCTTCTTGTGCGCATGAAGCG
**FMD4                         TCCCTGCCTCTTTCAACTTTGGCGCAATCCGGGCCACTGACATCAGTGAGCTTCTTGTGCGCATGAAGCG
**FMD6                         TCCCTGCCTCTTTCAACTTTGGTGCAATCCGGGCCACTGCCATTAGTGAGCTTCTTGTGCGCATGAAGCG
gi|380846218|gb|JF749848.1|    TCCCTGCCTCTTTCAACTTTGGTGCAATCCGGGCCACTGCCATTAGTGAGCTTCTTGTGCGCATGAAGCG
gi|304269208|gb|HM854022       TTCCTGCCTCCTTCAACTTTGGTGCAGTTCGAGCCACGACCATCCACGAGCTTCTCGTGCGCATGAAGCG
gi|332377434|gb|HQ832579       TTCCTGCCTCTTTCAACTTTGGTGCAGTTCAGGCCACTACCATCCACGAGCTGCTCGTGCGCATGAAGCG

                                      80        90       100       110       120       130       140
                               ....|....|....|....|....|....|....|....|....|....|....|....|....|....|
gi|1016883780|gb|KX002203.1|   GGCCGAACTCTACTGCCCTAGACCACTGTTGGCACTAGAGGTGATCTCACAAGACAGGCGCAAACAGAAG
gi|1016883774|gb|KX002200.1|   GGCCGAACTCTACTGCCCTAGACCACTGTTGGCACTAGAGGTGATCTCACAAGACAGGCACAAACAGAAG
gi|1016883753|gb|KX002188.1|   GGCCGAACTCTACTGCCCTAGACCACTGTTGGCACTAGAGGTGATCTCACAAGACAGGCACAAACAGAAG
gi|1016883773|gb|KX002199.1|   GGCCGAACTCTACTGCCCTAGACCACTGTTGGCACTAGAGGTGATCTCACAAGACAGGCACAAACAGAAG
gi|1016883764|gb|KX002194.1|   GGCCGAACTCTACTGCCCTAGACCACTGTTGGCACTAGAGGTGATCTCACAAGACAGGCACAAACAGAAG
gi|1016883757|gb|KX002190.1|   GGCCGAACTCTACTGCCCTAGACCACTGTTGGCACTAGAGGTGATCTCACAAGACAGGCACAAACAGAAG
gi|1016883766|gb|KX002195.1|   GGCCGAACTCTACTGCCCTAGACCACTGTTGGCACTAGAGGTGATCTCACAAGACAGGCACAAACAGAAG
gi|1016883770|gb|KX002197.1|   GGCCGAACTCTACTGCCCTAGACCACTGTTGGCACTAGAGGTGATCTCACAAGACAGGCACAAACAGAAG
gi|1016883782|gb|KX002204.1|   GGCCGAACTCTACTGCCCTAGACCACTGCTGGCACTAGAGGTGATCTCACAAGACAGACACAAACAGAAG
gi|1016883749|gb|KX002186.1|   AGCCGAACTCTACTGCCCTAGACCACTGCTGGCACTAGAGGTGATCTCACAAGACAGGCACAAACAGAAG
gi|1016883784|gb|KX002205.1|   GGCCGAACTCTACTGCCCTAGACCACTGTTGGCACTAGAGGTGATCTCACAAGACAGACACAAACAGAAG
gi|1016883751|gb|KX002187.1|   GGCCGAACTCTACTGCCCTAGACCACTGTTGGCACTAGAGGTGATCTCACAAGACAGACACAAACAGAAG
gi|1016883771|gb|KX002198.1|   GGCCGAACTCTACTGCCCTAGACCACTGTTGGCACTAGAGGTGATCTCACAAGACAGACACAAACAGAAG
gi|1016883760|gb|KX002192.1|   GGCCGAACTCTACTGCCCTAGACCACTGTTGGCACCAGAGGTGGTCTCACAAGACAGACACAAACAGAAG
gi|1016883778|gb|KX002202.1|   GGCCGAACTCTACTGCCCTAGACCACTGTTGGCACTAGAGGTGACCTTACAAGACAGGCACAAACAAAAG
gi|1016883768|gb|KX002196.1|   GGCCGAACTCTACTGCCCTAGACCACTGTTGGCACTAGAGGTGTCCTCACAAGACAGGCACAAACAGAAG
gi|1016883758|gb|KX002191.1|   GGCCGAACTCTACTGCCCTAGACCACTGTTGACACGAGAGGTGACTTCACAAGACAGGTACAAACAGGAG
**FMD1                         TGCTGAGCTCTACTGCCCTAGACCACTACTGGCAGTGGAAGTGACAGCCCAAGACAGGCACAAACAGAAG
gi|225032211|gb|FJ755018.1     TGCTGAGCTCTACTGCCCTAGACCACTACTGGCAGTGGAAGTGACAGCCCAAGACAGGCACAAACAGAAG
**FMD7                         TGCTGAGCTCTACTGCCCTAGACCACTACTGGCAGTGGAAGTGACAGCCCAAGACAGGCACAAACAGAAG
**FMD3                         TGCTGAGCTCTACTGCCCTAGACCACTACTGGCAGTGGAAGTCATAACCCAAGACAGGCACAAACAGAAG
gi|225032219|gb|FJ755022.1     TGCTGAGCTCTACTGCCCTAGACCACTACTGGCAGTGGAAGTCATAACCCAAGACAGGCACAAACAGAAG
gi|62083795|gb|AY960760.1|     TGCTGAGCTCTACTGCCCTAGACCACTACTGGCAGTGGAAGTGACAACCCAAGACAGGCACAAACAGAAG
**FMD2                         TGCTGAGCTCTACTGCCCTAGACCACTACTGGCAGTGGAAGTGACAACCCAAGACAGGCACAAACAGAAG
gi|126015403|gb|EF405982.1     TGCTGAGCTCTACTGCCCCAGGCCACTACTGGCAGTGGAGGTGACAGCGCAAGATAGGCACAAACAGAAG
**FMD5                         TGCTGAGCTCTACTGCCCCAGGCCACTACTGGCAGTGGAGGTGACAGCGCAAGATAGGCACAAACAGAAG
gi|46810838|gb|AY593791.1|     TGCTGAGCTCTACTGCCCCAGGCCACTACTGGCAGTGGAGGTGACAGCGCAAGATAGGCACAAACAGAAG
**FMD4                         TGCTGAGCTCTACTGCCCCAGGCCACTACTGGCAGTGGAGGTGACAGCGCAAGATAGGCACAAACAGAAG
**FMD6                         CGCTGAGCTCTACTGCCCTAGGCCACTACTGGCAGTGGAAGTGACAGAGCAGGACAGGCACAAACAGACG
gi|380846218|gb|JF749848.1|    CGCTGAGCTCTACTGCCCTAGGCCACTACTGGCAGTGGAAGTGACAGAGCAGGACAGGCACAAACAGACG
gi|304269208|gb|HM854022       TGCTGAGCTCTACTGCCCTAGACCACTGATGGCAGTGGAGGTGTCGTCACAAGACAGACACAAACAGAAG
gi|332377434|gb|HQ832579       TGCCGAACTCTACTGCCCCAGACCACTGTTGGCGGTGGAGGTCTCGTCGCAAGACAGACACAAACAGAAG

                                     150       160       170       180       190       200       210
                               ....|....|....|....|....|....|....|....|....|....|....|....|....|....|
gi|1016883780|gb|KX002203.1|   ATCATTGCACCCGAAAAACAGCTTTTGAATTTCGACCTGCTCAAGTTGGCGGGAGACGTTGAGTCCAACC
```

```
gi|1016883774|gb|KX002200.1|  ATCATTGCACCCGAAAAACAGCTTTTGAATTTCGACCTGCTCAAGTTGGCGGGAGACGTTGAGTCCAACC
gi|1016883753|gb|KX002188.1|  ATCATTGCACCCGAAAAACAGCTTTTGAATTTCGACCTGCTCAAGTTGGCGGGAGACGTTGAGTCCAACC
gi|1016883773|gb|KX002199.1|  ATCATTGCACCCGAAAAACAGCTTTTGAATTTCGACCTGCTCAAGTTGGCGGGAGACGTTGAGTCCAACC
gi|1016883764|gb|KX002194.1|  ATCATTGCACCCGAAAAACAGCTTTTGAATTTCGACCTGCTCAAGTTGGCGGGAGACGTTGAGTCCAACC
gi|1016883757|gb|KX002190.1|  ATCATTGCACCCGAAAAACAGCTTTTGAATTTCGACCTGCTCAAGTTGGCGGGAGACGTTGAGTCCAACC
gi|1016883766|gb|KX002195.1|  ATCATTGCACCCGAAAAACAGCTTTTGAATTTCGACCTGCTCAAGTTGGCGGGAGACGTTGAGTCCAACC
gi|1016883770|gb|KX002197.1|  ATCATTGCACCCGAAAAACAGCTTTTGAATTTCGACCTGCTCAAGTTGGCGGGAGACGTTGAGTCCAACC
gi|1016883782|gb|KX002204.1|  ATCATTGCACCCGAAAAACAGCTTTTGAATTTCGACCTGCTCAAGTTGGCGGGAGACGTTGAGTCCAACC
gi|1016883749|gb|KX002186.1|  ATCATTGCACCCGAAAAACAGCTTTTGAATTTCGACCTGCTCAAGTTGGCGGGAGACGTTGAGTCCAACC
gi|1016883784|gb|KX002205.1|  ATCATTGCACCCGAAAAACAGCTTTTGAATTTCGACCTGCTCAAGTTGGCGGGAGACGTTGAGTCCAACC
gi|1016883751|gb|KX002187.1|  ATCGTTGCACCCGAAAAACAGCTTTTGAATTTCGACCTGCTCAAGTTGGCGGGAGACGTTGAGTCCAACC
gi|1016883771|gb|KX002198.1|  ATCATTGCACCCGAAAAACAGCTTTTGAATTTCGACCTGCTCAAGTTGGCGGGAGACGTTGAGTCCAACC
gi|1016883760|gb|KX002192.1|  ATCATTGCACCCGAAAAACAGCTTTTGAATTTCGACCTGCTCAAGCTGGCGGGAGACGTTGAGTCCAACC
gi|1016883778|gb|KX002202.1|  ATCATTGCACCCGAAAAACAGCTTTTGAATTTCGACCTGCTCAAGTTGGCGGGAGACGTTGAGTCCAACC
gi|1016883768|gb|KX002196.1|  ATCATTGCACCCGAAAAACAGCTTTTGAATTTCGACCTGCTCAAGTTGGCGGGAGACGTTGAGTCCAACC
gi|1016883758|gb|KX002191.1|  ATCATTGCACCCGAAAAACAGCTTTTGAATTTCGACCTGCTCAAGTTGGCGGGGGACGTTGAGTCCAACC
**FMD1                        ATCATTGCGCCTGCGAAACAGCTCCTGAACTTTGACCTGCTCAAGTTGGCGGGAGACGTTGAGTCCAACC
gi|225032211|gb|FJ755018.1    ATCATTGCGCCTGCGAAACAGCTCCTGA------------------------------------------
**FMD7                        ATCATTGCGCCTGCGAAACAGCTCCTGAACTTTGACCTGCTCAAGTTGGCGGGAGACGTTGAGTCCAACC
**FMD3                        ATCATTGCGCCTGCGAAACAGCTCCTGAACTTTGACCTGCTCAAGTTGGCGGGAGACGTTGAGTCCAACC
gi|225032219|gb|FJ755022.1    ATCATTGCGCCTGCGAAACAGCTCCTGAACTTTGACCTGCTCAAGTTGGCGGGAGACGTTGAGTCCAACC
gi|62083795|gb|AY960760.1|    ATCATTGCGCCTGCGAAACAGCTCCTGAACTTTGACCTGCTCAAGTTGGCGGGAGACGTTGAGTCCAACC
**FMD2                        ATCATTGCGCCTGCGAAACAGCTCCTGAACTTTGACCTGCTCAAGTTGGCGGGAGACGTTGAGTCCAACC
gi|126015403|gb|EF405982.1    ATCATTGCGCCTGTGAAACAGCTCCTGAACTTTGACCTGCTCAAGTTGGCGGGAGACGTTGAGTCCAACC
**FMD5                        ATCATTGCGCCTGTGAAACAGCTCCTGAACTTTGACCTGCTCAAGTTGGCGGGAGACGTTGAGTCCAACC
gi|46810838|gb|AY593791.1|    ATCATTACGCCTGTGAAACAGCTCCTGAACTTTGACCTGCTCAAGTTGGCGGGAGACGTTGAGTCCAACC
**FMD4                        ATCATTACGCCTGTGAAACAGCTCCTGAACTTTGACCTGCTCAAGTTGGCGGGAGACGTTGAGTCCAACC
**FMD6                        ATCATTGCGCCTGCGAAGCAGCTCCTGAACTTTGACCTGCTCAAGTTGGCGGGAGACGTTGAGTCCAACC
gi|380846218|gb|JF749848.1|   ATCATTGCGCCTGCGAAGCAGCTCCTGAACTTTGACCTGCTCAAGTTGGCGGGAGACGTTGAGTCCAACC
gi|304269208|gb|HM854022      ATCATTGCACCAGCAAAACAGCTCCTGAACTTTGACCTGCTCAAGTTGGCGGGAGACGTTGAGTCCAACC
gi|332377434|gb|HQ832579      ATCATTGCACCAGCAAAACAGCTCCTGAACTTTGACCTGCTCAAGTTGGCAGGAGACGTTGAGTCCAACC

                                         220       230       240       250       260       270       280
                              ....|....|....|....|....|....|....|....|....|....|....|....|....|....|
gi|1016883780|gb|KX002203.1|  CTGGGCCCTTCTTCTTCTCCGACGTCAGGTCAAATTTCTCCAAGCTGGTGGAAACCATCAACCAGATGCA
gi|1016883774|gb|KX002200.1|  CTGGGCCCTTCTTCTTCTCCGACGTCAGGTCAAATTTCTCCAAGCTGGTGGAAACCATCAACCAGATGCA
gi|1016883753|gb|KX002188.1|  CTGGGCCCTTCTTCTTCTCCGACGTCAGGTCAAATTTCTCCAAGCTGGTGGAAACCATCAACCAGATGCA
gi|1016883773|gb|KX002199.1|  CTGGGCCCTTCTTCTTCTCCGACGTCAGGTCAAATTTCTCCAAGCTGGTGGAAACCATCAACCAGATGCA
gi|1016883764|gb|KX002194.1|  CTGGGCCCTTCTTCTTCTCCGACGTCAGGTCAAATTTCTCCAAGCTGGTGGAAACCATCAACCAGATGCA
gi|1016883757|gb|KX002190.1|  CTGGGCCCTTCTTCTTCTCCGACGTCAGGTCAAATTTCTCCAAGCTGGTGGAAACCATCAACCAGATGCA
gi|1016883766|gb|KX002195.1|  CTGGGCCCTTCTTCTTCTCCGATGTCAGGTCAAATTTCTCCAAGCTGGTGGAAACCATCAACCAGATGCA
gi|1016883770|gb|KX002197.1|  CTGGGCCCTTCTTCTTCTCCGACGTCAGGTCAAATTTCTCCAAGCTGGTGGAAACCATCAACCAGATGCA
gi|1016883782|gb|KX002204.1|  CTGGGCCTTTCTTCTTCTCCGACGTCAGGTCAAATTTCTCCAAGCTGGTGGAAACCATCAACCAGATGCA
gi|1016883749|gb|KX002186.1|  CTGGGCCCTTCTTCTTCTCCGACGTCAGGTCAAATTTCTCCAAGCTGGTGGAAACCATCAACCAGATGCA
gi|1016883784|gb|KX002205.1|  CTGGGCCCTTCTTCTTCTCCGACGTCAGGTCAAATTTCTCCAAGCTGGTGGAAACCATCAACCAGATGCA
gi|1016883751|gb|KX002187.1|  CTGGGCCCTTCTTCTTCTCCGACGTCAGGTCAAATTTCTCCAAGCTGGTGGAAACCATCAACCAGATGCA
gi|1016883771|gb|KX002198.1|  CTGGGCCCTTCTTCTTCTCCGACGTCAGGTCAAATTTCTCCAAGCTGGTGGAAACCATCAACCAGATGCA
gi|1016883760|gb|KX002192.1|  CTGGGCCCTTCTTCTTCTCCGACGTCAGGTCAAATTTCTCTAAGCTGGTGGAAACCATCAACCAGATGCA
gi|1016883778|gb|KX002202.1|  CTGGGCCCTTCTTCTTCTCCGACGTCAGGTCAAATTTCTCCAAGCTGGTGGAAACCATCAACCAGATGCA
gi|1016883768|gb|KX002196.1|  CTGGGCCCTTCTTCTTCTCCGACGTCAGGTCAAATTTCTCCAAGCTGGTGGAAACCGTCAACCAGATGCA
gi|1016883758|gb|KX002191.1|  CTGGGCCCTTCTTCTTCTCCGACGTCAGGTCAAATTTCTCCAAGCTGGTGGAAACCATCAACCAGATGCA
**FMD1                        CTGGGCCCTTCTTCTTCTCCGACGTCAGGACAAACTTCTCCAAACTGGTAGAGACCATCAACCAGATGCA
gi|225032211|gb|FJ755018.1    ----------------------------------------------------------------------
**FMD7                        CTGGGCCCTTCTTCTTCTCCGACGTCAGGACAAACTTCTCCAAACTGGTAGAGACCATCAACCAGATGCA
**FMD3                        CTGGGCCCTTCTTCTTCTCCGACGTCAGGACAAACTTCTCCAAACTGGTAGAGACCATCAACCAGATGCA
gi|225032219|gb|FJ755022.1    CTGGGCCCTTCTTCTTCTCCGACGTCAGGACAAACTTCTCCAAACTGGTAGAGACCATCAACCAGATGCA
gi|62083795|gb|AY960760.1|    CTGGGCCCTTCTTCTTCTCCGACGTCAGGACAAACTTCTCCAAGCTGGTAGAGACCATCAACCAGATGCA
**FMD2                        CTGGGCCCTTCTTCTTCTCCGACGTCAGGACAAACTTCTCCAAGCTGGTAGAGACCATCAACCAGATGCA
gi|126015403|gb|EF405982.1    CTGGGCCCTTCTTCTTCTCCGACGTCAGGACAAACTTCTCCAAAA-------------------------
**FMD5                        CTGGGCCCTTCTTCTTCTCCGACGTCAGGACAAACTTCTCCAAAATGGTAGAGACCATCAACCAGATGCA
gi|46810838|gb|AY593791.1|    CTGGGCCCTTCTTCTTCTCCGACGTCAGGACAAACTTCTCCAAACTGGTAGAGACCATCAACCAGATGCA
**FMD4                        CTGGGCCCTTCTTCTTCTCCGACGTCAGGACAAACTTCTCCAAACTGGTAGAGACCATCAACCAGATGCA
**FMD6                        CTGGGCCCTTCTTCTTCTCCGACGTCAGGACAAACTTCTCTAAACTGGTAGAGACCATTAACCAGATGCA
gi|380846218|gb|JF749848.1|   CTGGGCCCTTCTTCTTCTCCGACGTCAGGACAAACTTCTCTAAACTGGTAGAGACCATTAACCAGATGCA
gi|304269208|gb|HM854022      CTGGGCCCTTCTTCTTCTCTGACGTCAGGTCAAACTTCTCCAAACTGGTGGAGACCATCAACCAGATGCA
gi|332377434|gb|HQ832579      CTGGGCCCTTCTTCTTCTCCGACGTGAGGTCGAACTTCTCCAAACTGGTGGAAACCATCAACCAGATGCA

                                         290       300       310       320
                              ....|....|....|....|....|....|....|....|....|...
gi|1016883780|gb|KX002203.1|  GGAGGACATGTCAACAAAACACGGACCCGACTTTAACCGGTTGGTGTC
gi|1016883774|gb|KX002200.1|  GGAGGACATGTCAACAAAACACGGACCCGACTTTAACCGGTTGGTGTC
gi|1016883753|gb|KX002188.1|  GGAGGACATGTCAACAAAACACGGACCCGACTTTAACCGGTTGGTGTC
gi|1016883773|gb|KX002199.1|  GGAGGACATGTCAACAAAACACGGACCCGACTTTAACCGGTTGGTGTC
gi|1016883764|gb|KX002194.1|  GGAGGACATGTCAACAAAACACGGACCCGACTTTAACCGGTTGGTGTC
gi|1016883757|gb|KX002190.1|  GGAGGACATGTCAACAAAACACGGACCCGACTTTAACCGGTTGGTGTC
gi|1016883766|gb|KX002195.1|  GGAGGACATGTCAACAAAACACGGRCCCGACTTTAACCGGTTGGTGTC
gi|1016883770|gb|KX002197.1|  GGAGGACATGTCAACAAAACACGGACCCGACTTTAACCGGTTGGTGTC
```

```
gi|1016883782|gb|KX002204.1|  GGAGGACATGTCAACAAAACACGGACCCGACTTTAACCGGTTGGTGTC
gi|1016883749|gb|KX002186.1|  GGAGGACATGTCAACAAAACACGGACCCGACTTTAACCGGTTGGTGTC
gi|1016883784|gb|KX002205.1|  GGAGGACATGTCAACAAAACACGGACCCGACTTTAACCGGTTGGTGTC
gi|1016883751|gb|KX002187.1|  GGAGGACATGTCAACAAAACACGGACCCGACTTTAACCGGTTGGTGTC
gi|1016883771|gb|KX002198.1|  GGAGGACATGTCAACTAAACACGGACCCGACTTTAACCGGTTGGTGTC
gi|1016883760|gb|KX002192.1|  GGAGGACATGTCAACAAAACACGGACCCGACTTTAACCGGTTGGTGTC
gi|1016883778|gb|KX002202.1|  GGAGGACATGTCAACAAAACACGGACCCGACTTTAACCGGTTGGTGTC
gi|1016883768|gb|KX002196.1|  AGAGGACATGTCAACAAAACACGGACCCGACTTTAACCGGTTGGTGTC
gi|1016883758|gb|KX002191.1|  GGAGGACATGTCAACAAAACACGGACCCGACTTTAACCGGTTGGTGTC
**FMD1                        GGAGGACATGTCAACAAAACACGGACCCGACTTTAACCGGTTAGTTTC
gi|225032211|gb|FJ755018.1    ------------------------------------------------
**FMD7                        GGAGGACATGTCAACAAAACACGGACCCGACTTTAACCGGTTAGTTTC
**FMD3                        GGAGGACATGTCAACAAAACACGGACCCGACTTTAACCGGTTAGTTTC
gi|225032219|gb|FJ755022.1    GGAGGACATGTCAACAAAACACGGACCCGACTTTAACCGGTTAGTTTC
gi|62083795|gb|AY960760.1|    GGAGGACATGTCAACAAAACACGGACCCGACTTTAACCGGTTGGTCAC
**FMD2                        GGAGGACATGTCAACAAAACACGGACCCGACTTTAACCGGTTGGTCAC
gi|126015403|gb|EF405982.1    ------------------------------------------------
**FMD5                        GGAGGACATGTCAACAAAACACGGACCCGACTTTAACCGGTTAGTTTC
gi|46810838|gb|AY593791.1|    GGAGGACATGTCAACAAAACACGGACCCGACTTTAACCGGTTAGTTTC
**FMD4                        GGAGGACATGTCAACAAAACACGGACCCGACTTTAACCGGTTAGTTTC
**FMD6                        GGAGGATATGTCAACAAAACACGGACCCGACTTTAACCGGTTGGTTTC
gi|380846218|gb|JF749848.1|   GGAGGATATGTCAACAAAACACGGACCCGACTTTAACCGGTTGGTTTC
gi|304269208|gb|HM854022      AGAGGACATGTCAACAAAACACGGACCCGACTTCAACCGGTTGGTTTC
gi|332377434|gb|HQ832579      GGAGGACATGTCAACAAAACACGGACCCGACTTTAACCGGTTGGTGTC
```
