# Supplementary material for: Molecular and Serological Survey of Selected Viruses in Free-Ranging Wild Ruminants in Iran
Source: PLoS One. 2016 Dec 20;11(12):e0168756. doi: 10.1371/journal.pone.0168756 (PMC5173247; doi:10.1371/journal.pone.0168756)
Supplement: S3 File — GenBank accession numbers are shown at the left side of the figure and Iranian isolates are identified with double asterisk marks. (PDF) [file pone.0168756.s003.pdf]

Supplementary file 3: Nucleotide alignment of partial major glycoprotein B (gB) gene of Bovine herpesvirus 1. GenBank accession numbers are shown at the left side of the figure and Iranian isolates are identified with double asterisk marks.

```
                                    10        20        30        40        50        60        70
                            ....|....|....|....|....|....|....|....|....|....|....|....|....|....|
gi|562749910|gb|KF601565.1| AGAAGTGGCGCTGCCTTTCGAAAGCCGAGTACCTGCGCAGCGGGCGCAAGGTGGTGGCCTTTGACCGCGA
gi|614553405|gb|KF584168.1| AGAAGTGGCGCTGCCTTTCGAAAGCCGAGTACCTGCGCAGCGGGCGCAAGGTGGTGGCCTTTGACCGCGA
gi|658384413|gb|KJ652515.1| AGAAGTGGCGCTGCCTTTCGAAAGCCGAGTACCTGCGCAGCGGGCGCAAGGTGGTGGCCTTTGACCGCGA
gi|658384411|gb|KJ652514.1| AGAAGTGGCGCTGCCTTTCGAAAGCCGAGTACCTGCGCAGCGGGCGCAAGGTGGTGGCCTTTGACCGCGA
gi|658384421|gb|KJ652519.1| AGAAGTGGCGCTGCCTTTCGAAAGCCGAGTACCTGCGCAGCGGGCGCAAGGTGGTGGCCTTTGACCGCGA
gi|658384409|gb|KJ652513.1| AGAAGTGGCGCTGCCTTTCGAAAGCCGAGTACCTGCGCAGCGGGCGCAAGGTGGTGGCCTTTGACCGCGA
gi|658384417|gb|KJ652517.1| AGAAGTGGCGCTGCCTTTCGAAAGCCGAGTACCTGCGCAGCGGGCGCAAGGTGGTGGCCTTTGACCGCGA
gi|658384415|gb|KJ652516.1| AGAAGTGGCGCTGCCTTTCGAAAGCCGAGTACCTGCGCAGCGGGCGCAAGGTGGTGGCCTTTGACCGCGA
gi|658384419|gb|KJ652518.1| AGAAGTGGCGCTGCCTTTCGAAAGCCGAGTACCTGCGCAGCGGGCGCAAGGTGGTGGCCTTTGACCGCGA
gi|410060837|gb|JX898220.1| AGAAGTGGCGCTGCCTTTCGAAAGCCGAGTACCTGCGCAGCGGGCGCAAGGTGGTGGCCTTTGACCGCGA
**IBR-1                     AGAAGTGGCGCTGCCTTTCGAAAGCCGAGTACCTGCGCAGCGGGCGCAAGGTGGTGGCCTTTGACCGCGA
gi|960535267|gb|KU198480.1| AGAAGTGGCGCTGCCTTTCGAAAGCCGAGTACCTGCGCAGCGGGCGCAAGGTGGTGGCCTTTGACCGCGA

                                    80        90       100       110       120       130       140
                            ....|....|....|....|....|....|....|....|....|....|....|....|....|....|
gi|562749910|gb|KF601565.1| CGACGACCCCTGGGAGGCGCCGCTGAAGCCTGCGCGGCTGAGCGCGCCCGGGGTGCGGGGCTGGCACACG
gi|614553405|gb|KF584168.1| CGACGACCCCTGGGAGGCGCCGCTGAAGCCTGCGCGGCTGAGCGCGCCCGGGGTGCGGGGCTGGCACACG
gi|658384413|gb|KJ652515.1| CGACGACCCCTGGGAGGCGCCGCTGAAGCCTGCGCGGCTGAGCGCGCCCGGGGTGCGGGGCTGGCACACG
gi|658384411|gb|KJ652514.1| CGACGACCCCTGGGAGGCGCCGCTGAAGCCTGCGCGGCTGAGCGCGCCCGGGGTGCGGGGCTGGCACACG
gi|658384421|gb|KJ652519.1| CGACGACCCCTGGGAGGCGCCGCTGAAGCCTGCGCGGCTGAGCGCGCCCGGGGTGCGGGGCTGGCACACG
gi|658384409|gb|KJ652513.1| CGACGACCCCTGGGAGGCGCCGCTGAAGCCTGCGCGGCTGAGCGCGCCCGGGGTGCGGGGCTGGCACACG
gi|658384417|gb|KJ652517.1| CGACGACCCCTGGGAGGCGCCGCTGAAGCCTGCGCGGCTGAGCGCGCCCGGGGTGCGGGGCTGGCACACG
gi|658384415|gb|KJ652516.1| CGACGACCCCTGGGAGGCGCCGCTGAAGCCTGCGCGGCTGAGCGCGCCCGGGGTGCGGGGCTGGCACACG
gi|658384419|gb|KJ652518.1| CGACGACCCCTGGGAGGCGCCGCTGAAGCCTGCGCGGCTGAGCGCGCCCGGGGTGCGGGGCTGGCACACG
gi|410060837|gb|JX898220.1| CGACGACCCCTGGGAGGCGCCGCTGAAGCCTGCGCGGCTGAGCGCGCCCGGGGTGCGGGGCTGGCACACG
**IBR-1                     CGACGACCCCTGGGAGGCGCCGCTGAAGCCTGCGCGGCTGAGCGCGCCCGGGGTGCGGGGCTGGCACACG
gi|960535267|gb|KU198480.1| CGACGACCCCTGGGAGGCGCCGCTGAAGCCTGCGCGGCTGAGCGCGCCCGGGGTGCGGGGCTGGCACACG

                                   150       160       170       180       190       200       210
                            ....|....|....|....|....|....|....|....|....|....|....|....|....|....|
gi|562749910|gb|KF601565.1| ACGGACGATGTGTACACGGCGCTGGGCTCGGCGGGGCTCTACCGCACGGGCACCTCTGTGAACTGCATCG
gi|614553405|gb|KF584168.1| ACGGACGATGTGTACACGGCGCTGGGCTCGGCGGGGCTCTACCGCACGGGCACCTCTGTGAACTGCATCG
gi|658384413|gb|KJ652515.1| ACGGACGATGTGTACACGGCGCTGGGCTCGGCGGGGCTCTACCGCACGGGCACCTCTGTGAACTGCATCG
gi|658384411|gb|KJ652514.1| ACGGACGATGTGTACACGGCGCTGGGCTCGGCGGGGCTCTACCGCACGGGCACCTCTGTGAACTGCATCG
gi|658384421|gb|KJ652519.1| ACGGACGATGTGTACACGGCGCTGGGCTCGGCGGGGCTCTACCGCACGGGCACCTCTGTGAACTGCATCG
gi|658384409|gb|KJ652513.1| ACGGACGATGTGTACACGGCGCTGGGCTCGGCGGGGCTCTACCGCACGGGCACCTCTGTGAACTGCATCG
gi|658384417|gb|KJ652517.1| ACGGACGATGTGTACACGGCGCTGGGCTCGGCGGGGCTCTACCGCACGGGCACCTCTGTGAACTGCATCG
gi|658384415|gb|KJ652516.1| ACGGACGATGTGTACACGGCGCTGGGCTCGGCGGGGCTCTACCGCACGGGCACCTCTGTGAACTGCATCG
gi|658384419|gb|KJ652518.1| ACGGACGATGTGTACACGGCGCTGGGCTCGGCGGGGCTCTACCGCACGGGCACCTCTGTGAACTGCATCG
gi|410060837|gb|JX898220.1| ACGGACGATGTGTACACGGCGCTGGGCTCGGCGGGGCTCTACCGCACGGGCACCTCTGTGAACTGCATCG
**IBR-1                     ACGGACGATGTGTACACGGCGCTGGGCTCGGCGGGGCTCTACCGCACGGGCACCTCTGTGAACTGCATCG
gi|960535267|gb|KU198480.1| ACGGACGATGTGTACACGGCGCTGGGCTCGGCGGGGCTCTACCGCACGGGCACCTCTGTGAACTGCATCG

                                   220       230       240       250       260       270       280
                            ....|....|....|....|....|....|....|....|....|....|....|....|....|....|
gi|562749910|gb|KF601565.1| TGGAAGAAGTGGAGGCGCGCTCGGTGTACCCGTACGACTCGTTCGCGCTCTCGACCGGGGACATTATCTA
gi|614553405|gb|KF584168.1| TGGAAGAAGTGGAGGCGCGCTCGGTGTACCCGTACGACTCGTTCGCGCTCTCGACCGGGGACATTATCTA
gi|658384413|gb|KJ652515.1| TGGAAGAAGTGGAGGCGCGCTCGGTGTACCCGTACGACTCGTTCGCGCTCTCGACCGGGGACATTATCTA
gi|658384411|gb|KJ652514.1| TGGAAGAAGTGGAGGCGCGCTCGGTGTACCCGTACGACTCGTTCGCGCTCTCGACCGGGGACATTATCTA
gi|658384421|gb|KJ652519.1| TGGAAGAAGTGGAGGCGCGCTCGGTGTACCCGTACGACTCGTTCGCGCTCTCGACCGGGGACATTATCTA
gi|658384409|gb|KJ652513.1| TGGAAGAAGTGGAGGCGCGCTCGGTGTACCCGTACGACTCGTTCGCGCTCTCGACCGGGGACATTATCTA
gi|658384417|gb|KJ652517.1| TGGAAGAAGTGGAGGCGCGCTCGGTGTACCCGTACGACTCGTTCGCGCTCTCGACCGGGGACATTATCTA
gi|658384415|gb|KJ652516.1| TGGAAGAAGTGGAGGCGCGCTCGGTGTACCCGTACGACTCGTTCGCGCTCTCGACCGGGGACATTATCTA
gi|658384419|gb|KJ652518.1| TGGAAGAAGTGGAGGCGCGCTCGGTGTACCCGTACGACTCGTTCGCGCTCTCGACCGGGGACATTATCTA
gi|410060837|gb|JX898220.1| TGGAAGAAGTGGAGGCGCGCTCGGTGTACCCGTACGACTCGTTCGCGCTCTCGACCGGGGACATTATCTA
**IBR-1                     TGGAAGAAGTGGAGGCGCGCTCGGTGTACCCGTACGACTCGTTCGCGCTCTCGACCGGGGACATTATCTA
gi|960535267|gb|KU198480.1| TGGAAGAAGTGGAGGCGCGCTCGGTGTACCCGTACGACTCGTTCGCGCTCTCGACCGGGGACATTATCTA

                                   290       300       310       320       330       340       350
                            ....|....|....|....|....|....|....|....|....|....|....|....|....|....|
gi|562749910|gb|KF601565.1| CATGTCGCCCTTTTACGGGCTGCGCGAGGGCGCGCACCGCGAGCACACCAGCTACTCGCCGGAGCGCTTC
gi|614553405|gb|KF584168.1| CATGTCGCCCTTTTACGGGCTGCGCGAGGGCGCGCACCGCGAGCACACCAGCTACTCGCCGGAGCGCTTC
gi|658384413|gb|KJ652515.1| CATGTCGCCCTTTTACGGGCTGCGCGAGGGCGCGCACCGCGAGCACACCAGCTACTCGCCGGAGCGCTTC
gi|658384411|gb|KJ652514.1| CATGTCGCCCTTTTACGGGCTGCGCGAGGGCGCGCACCGCGAGCACACCAGCTACTCGCCGGAGCGCTTC
gi|658384421|gb|KJ652519.1| CATGTCGCCCTTTTACGGGCTGCGCGAGGGCGCGCACCGCGAGCACACCAGCTACTCGCCGGAGCGCTTC
gi|658384409|gb|KJ652513.1| CATGTCGCCCTTTTACGGGCTGCGCGAGGGCGCGCACCGCGAGCACACCAGCTACTCGCCGGAGCGCTTC
gi|658384417|gb|KJ652517.1| CATGTCGCCCTTTTACGGGCTGCGCGAGGGCGCGCACCGCGAGCACACCAGCTACTCGCCGGAGCGCTTC
gi|658384415|gb|KJ652516.1| CATGTCGCCCTTTTACGGGCTGCGCGAGGGCGCGCACCGCGAGCACACCAGCTACTCGCCGGAGCGCTTC
gi|658384419|gb|KJ652518.1| CATGTCGCCCTTTTACGGGCTGCGCGAGGGCGCGCACCGCGAGCACACCAGCTACTCGCCGGAGCGCTTC
gi|410060837|gb|JX898220.1| CATGTCGCCCTTTTACGGGCTGCGCGAGGGCGCGCACCGCGAGCACACCAGCTACTCGCCGGAGCGCTTC
**IBR-1                     CATGTCGCCCTTTTACGGGCTGCGCGAGGGCGCGCACCGCGAGCACACCAGCTACTCGCCGGAGCGCTTC
```

```
gi|960535267|gb|KU198480.1|  CATGTCGCCCTTTTACGGGCTGCGCGAGGGCGCGCACCGCGAGCACACCAGCTACTCGCCGGAGCGCTTC

                                     360       370       380       390       400       410       420
                             ....|....|....|....|....|....|....|....|....|....|....|....|....|....|
gi|562749910|gb|KF601565.1|  CAGCAGATCGAGGGCTACTACAAGCGCGACATGGCCACGGGCCGGCGCCTCAAGGAGCCGGTCTCGCGGA
gi|614553405|gb|KF584168.1|  CAGCAGATCGAGGGCTACTACAAGCGCGACATGGCCACGGGCCGGCGCCTCAAGGAGCCGGTCTCGCGGA
gi|658384413|gb|KJ652515.1|  CAGCAGATCGAGGGCTACTACAAGCGCGACATGGCCACGGGCCGGCGCCTCAAGGAGCCGGTCTCGCGGA
gi|658384411|gb|KJ652514.1|  CAGCAGATCGAGGGCTACTACAAGCGCGACATGGCCACGGGCCGGCGCCTCAAGGAGCCGGTCTCGCGGA
gi|658384421|gb|KJ652519.1|  CAGCAGATCGAGGGCTACTACAAGCGCGACATGGCCACGGGCCGGCGCCTCAAGGAGCCGGTCTCGCGGA
gi|658384409|gb|KJ652513.1|  CAGCAGATCGAGGGCTACTACAAGCGCGACATGGCCACGGGCCGGCGCCTCAAGGAGCCGGTCTCGCGGA
gi|658384417|gb|KJ652517.1|  CAGCAGATCGAGGGCTACTACAAGCGCGACATGGCCACGGGCCGGCGCCTCAAGGAGCCGGTCTCGCGGA
gi|658384415|gb|KJ652516.1|  CAGCAGATCGAGGGCTACTACAAGCGCGACATGGCCACGGGCCGGCGCCTCAAGGAGCCGGTCTCGCGGA
gi|658384419|gb|KJ652518.1|  CAGCAGATCGAGGGCTACTACAAGCGCGACATGGCCACGGGCCGGCGCCTCAAGGAGCCGGTCTCGCGGA
gi|410060837|gb|JX898220.1|  CAGCAGATCGAGGGCTACTACAAGCGCGACATGGCCACGGGCCGGCGCCTCAAGGAGCCGGTCTCGCGGA
**IBR-1                      CAGCAGATCGAGGGCTACTACAAGCGCGACATGGCCACGGGCCGGCGCCTCAAGGAGCCGGTCTCGCGGA
gi|960535267|gb|KU198480.1|  CAGCAGATCGAGGGCTACTACAAGCGCGACATGGCCACGGGCCGGCGCCTCAAGGAGCCGGTCTCGCGGA

                                     430       440
                             ....|....|....|....|....|....
gi|562749910|gb|KF601565.1|  ACTTTTTGCGTACACAGCACGTGACGGTA
gi|614553405|gb|KF584168.1|  ACTTTTTGCGTACACAGCACGTGACGGTA
gi|658384413|gb|KJ652515.1|  ACTTTTTGCGTACACAGCACGTGACGGTA
gi|658384411|gb|KJ652514.1|  ACTTTTTGCGTACACAGCACGTGACGGTA
gi|658384421|gb|KJ652519.1|  ACTTTTTGCGTACACAGCACGTGACGGTA
gi|658384409|gb|KJ652513.1|  ACTTTTTGCGTACACAGCACGTGACGGTA
gi|658384417|gb|KJ652517.1|  ACTTTTTGCGTACACAGCACGTGACGGTA
gi|658384415|gb|KJ652516.1|  ACTTTTTGCGTACACAGCACGTGACGGTA
gi|658384419|gb|KJ652518.1|  ACTTTTTGCGTACACAGCACGTGACGGTA
gi|410060837|gb|JX898220.1|  ACTTTTTGCGTACACAGCACGTGACGGTA
**IBR-1                      ACTTTTTGCGTACACAGCACGTGACGGTA
gi|960535267|gb|KU198480.1|  ACTTTTTGCGTACACAGCACGTGACGGTA
```
