# Supplementary material for: Molecular and Serological Survey of Selected Viruses in Free-Ranging Wild Ruminants in Iran
Source: PLoS One. 2016 Dec 20;11(12):e0168756. doi: 10.1371/journal.pone.0168756 (PMC5173247; doi:10.1371/journal.pone.0168756)
Supplement: S4 File — GenBank accession numbers are shown at the left side of the figure and Iranian isolates are identified with double asterisk marks. (PDF) [file pone.0168756.s004.pdf]

Supplementary file 4: Nucleotide alignment of partial tegument (teg) gene of Ovine herpesvirus 2. GenBank accession numbers are shown at the left side of the figure and Iranian isolates are identified with double asterisk marks.

```
                                       10        20        30        40        50        60        70
                                 ....|....|....|....|....|....|....|....|....|....|....|....|....|....|
gi|443496760|gb|KC123170.1|      CTGGGGTATATGAATCCAGATGGCTCTCGGTTAGGGTATCCGAAAGCAGCCCCAGTATCATGCTGACCCC
gi|331050|gb|L05908.1|HSVOVSE    CTGGGGTATATGAATCCAGATGGCTCTCGGTTAGGGTATCCGAAAGCAGCCCCAGTATCATGCTGACCCC
gi|410718442|gb|JQ780444.1|      CTGGGGGATATGAATCCAGATGGCTCTCGGTTAGGGTATCCGAAAGCAGCCCCAGTATCATGCTGACCCC
gi|757951587|gb|KJ658294.1|      CTGGGGGATATGAATCCAGATGGCTCTCGGTTAGGGTATCCGAAAGCAGCCCCAGTATCATGCTGACCCC
**OvHV-2.4                       CTGGGGGATATGAATCCAGATGGCTCTCGGTTAGGGTATCCGAAAGCAGCCCCAGTATCATGCTGACCCC
**OvHV-2.3                       CTGGGGGATATGAATCCAGATGGCTCTCGGTTAGGGTATCCGAAAGCAGCCCCAGTATCATGCTGACCCC
gi|558096295|gb|KF303529.1|      CTGGGGTATATGAATCCAGATGGCTCTCGGTTAGGGTATCCGAAAGCAGCCCCAGTATCATGCTGACCCC
gi|392843360|gb|JQ801454.1|      CTGGGGTATATGAATCCAGATGGCTCTCGGTTAGGGTATCCGAAAGCAGCCCCAGTATCATGCTGACCCC
gi|407080721|gb|JX442930.1|      CTGGGGTATATGAATCCAGATGGCTCTCGGTTAGGGTATCCGAAAGCAGCCCCAGTATCATGCTGACCCC
**OvHV-2.1                       CTGGGGTATATGAATCCAGATGGCTCTCGGTTAGGGTATCCGAAAGCAGCCCCAGTATCATGCTGACCCC
gi|756764242|gb|KP015737.1|      CTGGGGTATATGAATCCAGATGGCTCTCGGTTAGGGTATCCGAAAGCAGCCCCAGTATCATGCTGACCCC
gi|354620069|gb|JN084009.1|      CTGGGGTATATGAATCCAGATGGCTCTCGGTTAGGGTATCCGAAAGCAGCCCCAGTATCATGCTGACCCC
gi|354620069|gb|JN084009.1|      CTGGGGTATATGAATCCAGATGGCTCTCGGTTAGGGTATCCGAAAGCAGCCCCAGTATCATGCTGACCCC
gi|354620071|gb|JN084010.1|      -TGGGTTATATGAATCCAGATGGCTCTCGGTTAGGGTATCCGAAAGCAGCCCCAGTATCATGCTGACCCC
**OvHV-2.2                       CTGGGTTATATGAATCCAGATGGCTCTCGGTTAGGGTATCCGAAAGCAGCCCCAGTATCATGCTGACCCC
gi|339516600|gb|JF832385.1|      CCTGGGTATATGAATCCAGATGGCTCTCGGTTAGGGTATCCGAAAGCAGCCCCAGTATCATGCTGACCCC
gi|587656359|gb|KJ130037.1|      CCTGGGTATATGAATCCAGATGGCTCTCGGTTAGGGTATCCGAAAGCAGCCCCAGTATCATGCTGACCCC

                                       80        90       100       110       120       130       140
                                 ....|....|....|....|....|....|....|....|....|....|....|....|....|....|
gi|443496760|gb|KC123170.1|      TTGCAGAGGAATGGTCCTGCCTTGCTGGGTCCAGGGCACGTACCTGGGACTCTCTTACAGTGCGGACGGC
gi|331050|gb|L05908.1|HSVOVSE    TTGCAGAGGAATGGTCCTGCCTTGCTGGGTCCAGGGCACGTACCTGGGACTCTCTTACAGTGCGGACGGC
gi|410718442|gb|JQ780444.1|      TTGCAGAGGAATGGTCCTGCCTTGCTGGGTCCAGGGCACGTACCTGGGACTCTCTTACAGTGCGGACGGC
gi|757951587|gb|KJ658294.1|      TTGCAGAGGAATGGTCCTGCCTTGCTGGGTCCAGGGCACGTACCTGGGACTCTCTTACAGTGCGGACGGC
**OvHV-2.4                       TTGCAGAGGAATGGTCCTGCCTTGCTGGGTCCAGGGCAAATACCTGGGACTCTCTTACAGTGCGGACGGC
**OvHV-2.3                       TTGCAGAGGAATGGTCCTGCCTTGCTGGGTCCAGGGCAAATACCTGGGACTCTCTTACAGTGCGGACGGC
gi|558096295|gb|KF303529.1|      TTGCAGAGGAATGGTCCTGCCTTGCTGGGTCCAGGGCAAATACCTGGGACTCTCTTACAGTGCGGACGGC
gi|392843360|gb|JQ801454.1|      TTGCAGAGGAATGGTCCTGCCTTGCTGGGTCCAGGGCAAATACCTGGGACTCTCTTACAGTGCGGACGGC
gi|407080721|gb|JX442930.1|      TTGCAGAGGAATGGTCCTGCCTTGCTGGGTCCAGGGCAAATACCTGGGACTCTCTTACAGTGCGGACGGC
**OvHV-2.1                       TTGCAGAGGAATGGTCCTGCCTTGCTGGGTCCAGGGCAAATACCTGGGACTCTCTTACAGTGCGGACGGC
gi|756764242|gb|KP015737.1|      TTGCAGAGGAATGGTCCTGCCTTGCTGGGTCCAGGGCAAATACCTGGGACTCTCTTACAGTGCGGACGGC
gi|354620069|gb|JN084009.1|      TTGCAGAGGAATGGTCCTGCCTTGCTGGGTCCAGGGCAAATACCTGGGACTCTCTTACAGTGCGGACGGC
gi|354620069|gb|JN084009.1|      TTGCAGAGGAATGGTCCTGCCTTGCTGGGTCCAGGGCAAATACCTGGGACTCTCTTACAGTGCGGACGGC
gi|354620071|gb|JN084010.1|      TTGCAGAGGAATGGTCCTGCCTCGCTGGGTCCAGGGCAAATACCTGGGACTCTCTTACAGTGCGGACGGC
**OvHV-2.2                       TTGCAGAGGAATGGTCCTGCCTCGCTGGGTCCAGGGCAAATACCTGGGACTCTCTTACAGTGCGGACGGC
gi|339516600|gb|JF832385.1|      TTGCAGAGGAATGGTCCTGCCTTGCTGGGTCCAGGGCAAATACCTGGGACTCTCTTACAGTGCGGACGGC
gi|587656359|gb|KJ130037.1|      TTGCAGAGGAATGGTCCTGCCTTGCTGGGTCCAGGGCAAATACCTGGGACTCTCTTACAGTGCGGACGGC

                                      150       160       170       180       190       200       210
                                 ....|....|....|....|....|....|....|....|....|....|....|....|....|....|
gi|443496760|gb|KC123170.1|      GTGGAGCACAGTTTATTTCAGACAAACACAGTAGCCTGCTCCTACCACGGTCAGTCCAAGACCCCCGAAG
gi|331050|gb|L05908.1|HSVOVSE    GTGGAGCACAGTTTATTTCAGACAAACACGGTAGCCTGCTCCTACCACGGTCAGTCCAAGACCCCCGAAG
gi|410718442|gb|JQ780444.1|      GTGGAGCACAGTTTATTTCAGACAAACACGGTAGCCTGCTCCTACCACGGTCAGTCCAAGACCCCCGAAG
gi|757951587|gb|KJ658294.1|      GTGGAGCACAGTTTATTTCAGACAAACACGGTAGCCTGCTCCTACCACGGTCAGTCCAAGACCCCCGAAG
**OvHV-2.4                       GTGGAGCACAGTTTATTTCAGACAAACACGGTAGCCTGCTCCTACCACGGTCAGTCCAAGACCCCCGAAG
**OvHV-2.3                       GTGGAGCACAGTTTATTTCAGACAAACACGGTAGCCTGCTCCTACCACGGTCAGTCCAAGACCCCCGAAG
gi|558096295|gb|KF303529.1|      GTGGAGCACAGTTTATTTCAGACAAACACGGTATCCTGCTCCTACCACGGTCAGTCCAAGACCCCCGAAG
gi|392843360|gb|JQ801454.1|      GTGGAGCACAGTTTATTTCAGACAAACACGGTAGCCTGCTCCTACCACGGTCAGTCCAAGGCCCCCGAAG
gi|407080721|gb|JX442930.1|      GTGGAGCACAGTTTATTTCAGACAAACACGGTAGCCTGCTCCTACCACGGTCAGTCCAAGACCCCCGAAG
**OvHV-2.1                       GTGGAGCACAGTTTATTTCAGACAAACACGGTAGCCTGCTCCTACCACGGTCAGTCCAAGACCCCCGAAG
gi|756764242|gb|KP015737.1|      GTGGAGCACAGTTTATTTCAGACAAACACGGTAGCCTGCTCCTACCACGGTCAGTCCAAGACCCCCGAAG
gi|354620069|gb|JN084009.1|      GTGGAGCACAGTTTATTTCAGACAAACACGGTAGCCTGCTCCTACCACGGTCAGTCCAAGACCCCCGAAG
gi|354620069|gb|JN084009.1|      GTGGAGCACAGTTTATTTCAGACAAACACGGTAGCCTGCTCCTACCACGGTCAGTCCAAGACCCCCGAAG
gi|354620071|gb|JN084010.1|      GTGGAGCACAGTTTATTTCAGACAAACACGGTAGCCTGCTCCTACCACGGTCAGTCTAAGAC--------
**OvHV-2.2                       GTGGAGCACAGTTTATTTCAGACAAACACGGTAGCCTGCTCCTACCACGGTCAGTCTAAGACCCCCGAAG
gi|339516600|gb|JF832385.1|      GTGGAGCACAGTTTATTTCAGACAAACACGGTAGCCTGCTCCTACCACGGTCAGTCCAAGACCCCCGAAG
gi|587656359|gb|KJ130037.1|      GTGGAGCACAGTTTATTTCAAACAAACACGGTAGCCTGCTCCTACCACGGTCAGTCCAAGACCCCCGAAG

                                      220       230
                                 ....|....|....|....|....
gi|443496760|gb|KC123170.1|      CCTTCGCTCGCCACTACCCCAGAA
gi|331050|gb|L05908.1|HSVOVSE    CCTTCGCTCGCCACTACCCCAGAA
gi|410718442|gb|JQ780444.1|      CCTTCGCTCGCCACTACCCCAGAA
gi|757951587|gb|KJ658294.1|      CCTTCGCTCGCCACTACCCCAGAA
**OvHV-2.4                       CCTTCGCTCGCCACTACCCCAGAA
**OvHV-2.3                       CCTTCGCTCGCCACTACCCCAGAA
gi|558096295|gb|KF303529.1|      CCTTCGCTCGCCACTACCCCAGAA
gi|392843360|gb|JQ801454.1|      CCTTCGCTCGCCACTACCCCAGAA
gi|407080721|gb|JX442930.1|      CCTTCGCTCGCCACTACCCCAGAA
**OvHV-2.1                       CCTTCGCTCGCCACTACCCCAGAA
gi|756764242|gb|KP015737.1|      CCTTCGCTCGCCACTACCCCAGAA
```

```
gi|354620069|gb|JN084009.1|    CCTTCGCTCGCCACTACCCCAGAA
gi|354620069|gb|JN084009.1|    CCTTCGCTCGCCACTACCCCAGAA
gi|354620071|gb|JN084010.1|    ------------------------
**OvHV-2.2                     CCTTCGCTCGCCACTACCCCAGAA
gi|339516600|gb|JF832385.1|    CCTTCGCTCGCCACTACCCCAGAA
gi|587656359|gb|KJ130037.1|    CCTTCGCTCGCCACTACCCCAGAA
```
