# Supplementary material for: Molecular and Serological Survey of Selected Viruses in Free-Ranging Wild Ruminants in Iran
Source: PLoS One. 2016 Dec 20;11(12):e0168756. doi: 10.1371/journal.pone.0168756 (PMC5173247; doi:10.1371/journal.pone.0168756)
Supplement: S5 File — GenBank accession numbers are shown at the left side of the figure and Iranian isolates are identified with double asterisk marks. (PDF) [file pone.0168756.s005.pdf]

Supplementary file 5: Nucleotide alignment of partial nucleoprotein (N) gene of Peste-des-petits-ruminants virus. GenBank accession numbers are shown at the left side of the figure and Iranian isolates are identified with double asterisk marks.

```
                                  10        20        30        40        50        60        70
                          ....|....|....|....|....|....|....|....|....|....|....|....|....|....|
gi|110341787|gb|DQ837640.1|  GGGGACGAAAGAACCGCTAGAGGGACTGGGCCTCGACAGGCGCAGGTCTCCTTCCTCCAGCATAAAATAA
gi|110341775|gb|DQ840194.1|  GGGGACGAAAGAACCGCTAGAGGGACTGGGCCTCGACAGGCGCAGGTCTCCTTCCTCCAGCGCAAAATAG
gi|307646466|gb|HQ131938.1|  GGGGACGAAAGAACCGCTAGAGGGACTGGGCCTCGACAGGCGCAGGTCTCCTTCCTCCAGCACAAAGCAG
gi|307646464|gb|HQ131937.1|  GGGGACGAAAGAACCGCTAGAGGGACTGGGCCTCGACAGGCGCAGGTCTCCTTCCTCCAGCACAAAGCAG
gi|110341777|gb|DQ840195.1|  GGGGACGAAAGAACCGCTAGAGGGACTGGGCCTCGACAGGCGCAGGTCTCCTTCCTCCAGCACAAAGCAG
gi|110341761|gb|DQ840187.1|  GGGGACGAAAGAACCGCTAGAGGGACTGGGCCTCGACAGGCGCAGGTCTCCTTCCTCCAGCACAAAACAG
gi|110341757|gb|DQ840185.1|  GGGGACGAAAGAACCGCTAGAGGGACTGGGCCTCGACAGGCGCAGGTCTCCTTCCTCCAGCACAAAACAG
gi|110341783|gb|DQ840198.1|  GGGGACGAAAGAACCGCTAGAGGGACTGGGCCTCGACAGGCGCAGGTCTCCTTCCTCCAGCACCAAACAG
gi|110341781|gb|DQ840197.1|  GGGGACGAAAGAACCGCTAGAGGGACTGGGCCTCGACAGGCGCAGGTCTCCTTCCTCCAGCACAAAACAG
gi|110341779|gb|DQ840196.1|  GGGGACGAAAGAACCGCTAGAGGGACTGGGCCTCGACAGGCGCAGGTCTCCTTCCTCCAGCACAAAACAG
gi|431837877|gb|JX898859.1|  GGGGACGAAAGAACCGCTAGAGGGACTGGGCCTCGACAGGCGCAGGTCTCCTTCCTCCAGCACAAAACAG
gi|431837883|gb|JX898862.1|  GGGGACGAAAGAACCGCTAGAGGGACTGGGCCTCGACAGGCGCAGGTCTCCTTCCTCCAGCACAAAACAG
gi|431837857|gb|JX898849.1|  GGGGACGAAAGAACCGCTAGAGGGACTGGGCCTCGACAGGCGCAGGTCTCCTTCCTCCAGCACAAAACAG
gi|431837853|gb|JX898847.1|  GGGGACGAAAGAACCGCTAGAGGGACTGGGCCTCGACAGGCGCAGGTCTCCTTCCTCCAGCACAAAACAG
gi|431837873|gb|JX898857.1|  GGGGACGAAAGAACCGCTAGAGGGACTGGGCCTCGACAGGCGCAGGTCTCCTTCCTCCAGCACAAAACAG
gi|431837865|gb|JX898853.1|  GGGGACGAAAGAACCGCTAGAGGGACTGGGCCTCGACAGGCGCAGGTCTCCTTCCTCCAGCACAAAACAG
gi|431837861|gb|JX898851.1|  GGGGACGAAAGAACCGCTAGAGGGACTGGGCCTCGACAGGCGCAGGTCTCCTTCCTCCAGCACAAAACAG
gi|431837867|gb|JX898854.1|  GGGGACGAAAGAACCGCTAGAGGGACTGGGCCTCGACAGGCGCAGGTCTCCTTCCTCCAGCACAAAACAG
gi|431837863|gb|JX898852.1|  GGGGACGAAAGAACCGCTAGAGGGACTGGGCCTCGACAGGCGCAGGTCTCCTTCCTCCAGCACAAAACAG
gi|431837859|gb|JX898850.1|  GGGGACGAAAGAACCGCTAGAGGGACTGGGCCTCGACAGGCGCAGGTCTCCTTCCTCCAGCACAAAACAG
gi|431837855|gb|JX898848.1|  GGGGACGAAAGAACCGCTAGAGGGACTGGGCCTCGACAGGCGCAGGTCTCCTTCCTCCAGCACAAAACAG
gi|431837869|gb|JX898855.1|  GGGGACGAAAGAACCGCTAGAGGGACTGGGCCTCGACAGGCGCAGGTCTCCTTCCTCCAGCACAAAACAG
**PPR5                       GGGGACGAAAGAACCGCTAGAGGGACTGGGCCTCGACAGGCGCAGGTCTCCTTCCTCCAGCACAAAACAG
**PPR4                       GGGGACGAAAGAACCGCTAGAGGGACTGGGCCTCGACAGGCGCAGGTCTCCTTCCTCCAGCACAAAACAG
gi|431837871|gb|JX898856.1|  GGGGACGAAAGAACCGCTAGAGGGACTGGGCCTCGACAGGCGCAGGTCTCCTTCCTCCAGCACAAAACAG
**PPR3                       GGGGACGAAAGAACCGCTAGAGGGACTGGGCCTCGACAGGCGCAGGTCTCCTTCCTCCAGCACAAAACAG
gi|431837875|gb|JX898858.1|  GGGGACGAAAGAACCGCTAGAGGGACTGGGCCTCGACAGGCGCAGGTCTCCTTCCTCCAGCACAAAACAG
**PPR1                       GGGGACGAAAGAACCGCTAGAGGGACTGGGCCTCGACAGGCGCAGGTCTCCTTCCTCCAGCACAAAACAG
gi|431837881|gb|JX898861.1|  GGGGACGAAAGAACCGCTAGAGGGACTGGGCCTCGACAGGCGCAGGTCTCCTTCCTCCAGCACAAAACAG
gi|431837879|gb|JX898860.1|  GGGGACGAAAGAACCGCTAGAGGGACTGGGCCTCGACAGGCGCAAGTCTCCTTCCTCCAGCACAAAACAG
**PPR2                       GGGGACGAAAGAACCGCTAGAGGGACTGGGCCTCGACAGGCGCAGGTCTCCTTCCTCCAGCACAAAACAG
gi|110341785|gb|DQ840199.1|  GGGGACGAAAGAACCGCTAGAGGGACTGGGCCTCGACAGGCGCAGGTCTCTTTCCTCCAGCACAAAACAG

                                  80        90       100       110       120       130       140
                          ....|....|....|....|....|....|....|....|....|....|....|....|....|....|
gi|110341787|gb|DQ837640.1|  ATGAGGGAGAGTCGCCTACACCAGCGACCAGAGAAGAAGTCAAAGCTGCGATCCCAAATGGGTCTGAAGG
gi|110341775|gb|DQ840194.1|  GTGAGGGAGAGTCGCCTACACCAGCGACCAAAGAAGAAGTCAAAGCTGCGATCCCAAACGGGTCTGAAGG
gi|307646466|gb|HQ131938.1|  GAGAGGGAGATTCGCCCGCACCAGCGACCAGAGAAGGGGTCAAAGCTGCGATCTCAAACGGATCTGAAGA
gi|307646464|gb|HQ131937.1|  GAGAGGGAGATTCGCCCGCACCAGCGACCAGAGAAGGGGTCAAAGCTGCGATCTCAAACGGATCTGAAGA
gi|110341777|gb|DQ840195.1|  GAGAGGGAGATTCGCCCGCACCAGCGACCAGAGAAGGGGTCAAAGCTGCGATCTCAAACGGATCTGAAGA
gi|110341761|gb|DQ840187.1|  GAGAAGGAGATTCGCCCGCACCAGCGACCAGAGAAGGGGTCAAAGCTGCGATCTCAAACGGATCTGAAGA
gi|110341757|gb|DQ840185.1|  GAGAAGGAGATTCGCCCGCACCAGCGACCAGAGAAGGGGTCAAAGCTGCGATCTCAAACGGATCTGAAGA
gi|110341783|gb|DQ840198.1|  GAGGGGGAGAGTCGTCCGCACCAGCGACCAGAGAAGGGGTCAAAGCTGCGATCCCAAACGGATCTGAAGA
gi|110341781|gb|DQ840197.1|  TAGAGGGGGAGTCGTCCGCACCAGCGACCAGAGAAGGGGTCAAAGCTGCGATCCCAAACGGATCTGAAGA
gi|110341779|gb|DQ840196.1|  TAGAGGGGGAGTCGTCCGCACCAGCGACCAGAGAAGGGGTCAAAGCTGCGATCCCAAACGGATCTGAAGA
gi|431837877|gb|JX898859.1|  GAGAGGGAGAGTCGTCCGCACCAGCAACCAGAGAGGGGGTCAAAGCTGCGATCCCAAACGGATCCGAAGA
gi|431837883|gb|JX898862.1|  GAGAGGGAGAGTCGTCCGCACCAGCAACCAGAGAGGGGGTCAAAGCTGCGATCCCAAACGGATCCGAAGA
gi|431837857|gb|JX898849.1|  GAGAGGGAGAGTCGTCCGCACCAGCAACCAGAGAGGGGGTCAAAGCTGCGATCCCAAACGGATCCGAAGA
gi|431837853|gb|JX898847.1|  GAGAGGGAGAGTCGTCCGCACCAGCAACCAGAGAGGGGGTCAAAGCTGCGATCCCAAACGGATCCGAAGA
gi|431837873|gb|JX898857.1|  GAGAGGGAGAGTCGTCCGCACCAGCAACCAGAGAGGGGGTCAAAGCTGCGATCCCAAACGGATCCGAAGA
gi|431837865|gb|JX898853.1|  GAGAGGGAGAGTCGTCCGCACCAGCAACCAGAGAGGGGGTCAAAGCTGCGATCCCAAACGGATCCGAAGA
gi|431837861|gb|JX898851.1|  GAGAGGGAGAGTCGTCCGCACCAGCAACCAGAGAGGGGGTCAAAGCTGCGATCCCAAACGGATCCGAAGA
gi|431837867|gb|JX898854.1|  GAGAGGGAGAGTCGTCCGCACCAGCAACCAGAGAGGGGGTCAAAGCTGCGATCCCAAACGGATCCGAAGA
gi|431837863|gb|JX898852.1|  GAGAGGGAGAGTCGTCCGCACCAGCAACCAGAGAGGGGGTCAAAGCTGCGATCCCAAACGGATCCGAAGA
gi|431837859|gb|JX898850.1|  GAGAGGGAGAGTCGTCCGCACCAGCAACCAGAGAGGGGGTCAAAGCTGCGATCCCAAACGGATCCGAAGA
gi|431837855|gb|JX898848.1|  GAGAGGGAGAGTCGTCCGCACCAGCAACCAGAGAGGGGGTCAAAGCTGCGATCCCAAACGGATCCGAAGA
gi|431837869|gb|JX898855.1|  GAGAGGGAGAGTCGTCCGCACCAGCAACCAGAGAGGGGGTCAAAGCTGCGATCCCAAACGGATCCGAAGA
**PPR5                       GAGAGGGAGAGTCGTCCGCACCAGCAACCAGAGAGGGGGTCAAAGCTGCGATCCCAAACGGATCCGAAGA
**PPR4                       GAGAGGGAGAGTCGTCCGCACCAGCAACCAGAGAGGGGGTCAAAGCTGCGATCCCAAACGGATCCGAAGA
gi|431837871|gb|JX898856.1|  GAGAGGGAGAGTCGTCCGCACCAGCAACCAGAGAGGGGGTCAAAGCTGCGATCCCAAACGGATCCGAAGA
**PPR3                       GAGAGGGAGAGTCGTCCGCACCAGCAACCAGAGAAGGGGTCAAAGCTGCGATCCCACACGGATCCGAAGA
gi|431837875|gb|JX898858.1|  GAGAGGGAGAGTCGTCCGCACCAGCAACCAGAGAAGGGGTCAAAGCTGCGATCCCACACGGATCCGAAGA
**PPR1                       GAGAGGGAGAGTCGTCCGCACCAGCAACCAGAGAAGGGGTCAAAGCTGCGATCCCAAACGGATCCGAAGA
gi|431837881|gb|JX898861.1|  GAGAGGGAGAGTCGTCCGCACCAGCAACCAGAGAAGGGGTCAAAGCTGCGATCCCAAACGGATCCGAAGA
gi|431837879|gb|JX898860.1|  GAGAGGGAGAGTCGTCCGCACCAGCAACCAGAGAAGGGGTCAAAGCTGCGATCCCAAACGGATCCGAAGA
**PPR2                       TAGAGGGAGAGTCGTCCGCACCAGCAACCAGAGAAGGGGTCAAAGCTGCGATCCCAAACGGATCCGAAGA
gi|110341785|gb|DQ840199.1|  GAGAGGGAGAGTCATCTGCACCAGTGACCAGAGAAGAAGTCAAGGCTGCGATTCCGAATGGGTCCGAAGA

                                 150       160       170       180       190       200       210
                          ....|....|....|....|....|....|....|....|....|....|....|....|....|....|
```

```
gi|110341787|gb|DQ837640.1|  AAGGGACATAAAGCGAACACGCTCAGGAAAGCCCAGAGGAGAAACTTCCGGCCAACTGCTTCTGGAGATC
gi|110341775|gb|DQ840194.1|  AAGGGACATGAAGCGAACACGCCCAGGGAAGCCCAGAGGAGAAACTCCCGGCCAACTGCTTCTGGAGATC
gi|307646466|gb|HQ131938.1|  AAGGGACAGAAAGCAAACACGCCCAGGAAGGCCCAGAGGAGAGACCCCCGGTCAACTGCTCCTGGAAATC
gi|307646464|gb|HQ131937.1|  AAGGGACAGAAAGCAAACACGCCCAGGAAGGCCCAGAGGAGAGACCCCCGGTCAACTGCTCCTGGAAATC
gi|110341777|gb|DQ840195.1|  AAGGGACAGAAAGCAAACACGCCCAGGAAGGCCCAGAGGAGAGACCCCCGGTCAACTGCTCCTGGAAATC
gi|110341761|gb|DQ840187.1|  AAGGGACAGAAAGCAAACACGCCCAGGAAGGCCCAGAGGAGAGAGCCCCGGCCAACTGCTCCTGGAAATC
gi|110341757|gb|DQ840185.1|  AAGGGACAGAAAGCAAACACGCCCAGGAAGGCCCAGAGGAGAGAGCCCCGGCCAACTGCTCCTGGAAATC
gi|110341783|gb|DQ840198.1|  AAGGGACAGAAAGCAAACACGCCCAGGAAGGCCCAGAGGAGAGACCCCCGGCCAACTGCTCCTGGACATC
gi|110341781|gb|DQ840197.1|  AAGGGACAGAAAGCAAACACGCCCAGGAAGGCCCAGAGGAGAGACCCCCGGCCAACTGCTCCTGGAAATC
gi|110341779|gb|DQ840196.1|  AAGGGACAGAAAGCAAACACGCCCAGGAAGGCCCAGAGGAGAGACCCCCGGCCAACTGCTCCTGGAAATC
gi|431837877|gb|JX898859.1|  AAGGGACAGGAAGCAAACACGCTCAGGAAGGCCCAGAGGAGAGACCCCCAGCCAACTGCTCCTGGAAATC
gi|431837883|gb|JX898862.1|  AAGGGACAGGAAGCAAACACGCTCAGGAAGGCCCAGAGGAGAGACCCCCAGCCAACTGCTCCTGGAAATC
gi|431837857|gb|JX898849.1|  AAGGGACAGGAAGCAAACACGCTCAGGAAGGCCCAGAGGAGAGACCCCCAGCCAACTGCTCCTGGAAATC
gi|431837853|gb|JX898847.1|  AAGGGACAGGAAGCAAACACGCTCAGGAAGGCCCAGAGGAGAGACCCCCAGCCAACTGCTCCTGGAAATC
gi|431837873|gb|JX898857.1|  AAGGGACAGGAAGCAAACACGCTCAGGAAGGCCCAGAGGAGAGACCCCCAGCCAACTGCTCCTGGAAATC
gi|431837865|gb|JX898853.1|  AAGGGACAGGAAGCAAACACGCTCAGGAAGGCCCAGAGGAGAGACCCCCAGCCAACTGCTCCTGGAAATC
gi|431837861|gb|JX898851.1|  AAGGGACAGGAAGCAAACACGCTCAGGAAGGCCCAGAGGAGAGACCCCCAGCCAACTGCTCCTGGAAATC
gi|431837867|gb|JX898854.1|  AAGGGACAGGAAGCAAACACGCTCAGGAAGGCCCAGAGGAGAGACCCCCAGCCAACTGCTCCTGGAAATC
gi|431837863|gb|JX898852.1|  AAGGGACAGGAAGCAAACACGCTCAGGAAGGCCCAGAGGAGAGACCCCCAGCCAACTGCTCCTGGAAATC
gi|431837859|gb|JX898850.1|  AAGGGACAGGAAGCAAACACGCTCAGGAAGGCCCAGAGGAGAGACCCCCAGCCAACTGCTCCTGGAAATC
gi|431837855|gb|JX898848.1|  AAGGGACAGGAAGCAAACACGCTCAGGAAGGCCCAGAGGAGAGACCCCCAGCCAACTGCTCCTGGAAATC
gi|431837869|gb|JX898855.1|  AAGGGACAGGAAGCAAACACGCTCAGGAAGGCCCAGAGGAGAGACCCCCAGCCAACTGCTCCTGGAAATC
**PPR5                       AAGGGACAGGAAGCAAACACGCTCAGGAAGGCCCAGAGGAGAGACCCCCAGCCAACTGCTCCTGGAAATC
**PPR4                       AAGGGACAGGAAGCAAACACGCTCAGGAAGGCCCAGAGGAGAGACCCCCAGCCAACTGCTCCTGGAAATC
gi|431837871|gb|JX898856.1|  AAGGGACAGGAAGCAAACACGCTCAGGAGGGCCCAGAGGAGAGACCCCCAGCCAACTGCTCCTGGAAATC
**PPR3                       AAGGGACAGGAAGCAAACACGCTCAGGAAGGCCCAGAGGAGAGACCCCCAGCCAACTGCTCCCGGAAATC
gi|431837875|gb|JX898858.1|  AAGGGACAGGAAGCAAACACGCTCAGGAAGGCCCAGAGGAGAGACCCCCAGCCAACTGCTCCCGGAAATC
**PPR1                       AAGGGACAGGAAGCAAACACGCTCAGGAAGGCCCAGAGGAGAGACCCCCAGCCAACTGCTCCTGGAAATC
gi|431837881|gb|JX898861.1|  ACGGGACAGAAAGCAAACACGCCCAGGAAGGTCCAGAGGAGAGACCCCCAGCCAACTGCTCCTGGAAATC
gi|431837879|gb|JX898860.1|  ACGGGACAGAAAGCAAACACGCCCAGGAAGGTCCAGAGGAGAGACCCCCAGCCAACTGCTCCTGGAAATC
**PPR2                       AAGGGACAGAAAGCAAACACGCCCAGGAAGGCCCAGAGGAGAGACCCCCGGCCAACTGCTCCTGGAAATC
gi|110341785|gb|DQ840199.1|  AAGGGACAAAAGGCGAACCCGCCCAGGAAGGTCCAGAGGAGAAATCCCGAGCCAACCTCTCCTGGAAATC

                                       220       230       240       250       260       270       280
                             ....|....|....|....|....|....|....|....|....|....|....|....|....|....|
gi|110341787|gb|DQ837640.1|  ATGCCAGAGGATGAAGTCTCGCGAGAGTCTAGTCAAAACCCTCGTGAGGCTCAAAGATCGGCTGAGGCAC
gi|110341775|gb|DQ840194.1|  ATGCCAGAGGATGAAGTCTCGCGAGAGTCTGGTCAAAACCCTCGTGAGGCTCAAAGATCGGCTGAGGCAC
gi|307646466|gb|HQ131938.1|  ATGCCAGAGGATGAGGTCTCGCGAGAGTCTGGTCAAAACCCTCGTGAGGCTCAAAGATCAGCCGAGGCAC
gi|307646464|gb|HQ131937.1|  ATGCCAGAGGATGAGGTCTCGCGAGAGTCTGGTCAAAACCCTCGTGAGGCTCAAAGATCAGCCGAGGCAC
gi|110341777|gb|DQ840195.1|  ATGCCAGAGGATGAGGTCTCGCGAGAGTCTGGTCAAAACCCTCGTGAGGCTCAAAGATCAGCCGAGGCAC
gi|110341761|gb|DQ840187.1|  ATGCCAGAGGATGAGGTCTCGCGAGAGTCTGGTCAAAACCCTCGTGAGGCTCAAAGATCGGCCGAGGCAC
gi|110341757|gb|DQ840185.1|  ATGCCAGAGGATGAGGTCTCGCGAGAGTCTGGTCAAAACCCTCGTGAGGCTCAAAGATCGGCCGAGGCAC
gi|110341783|gb|DQ840198.1|  ATGCCAGAGGATGAGGTCTCGCGAGAGTCTGGTCAAAACCCTCGTGAGGCTCAAAGGTCAGCCGAGGCAC
gi|110341781|gb|DQ840197.1|  ATGCCAGAGGATGAGGTCTCGCGAGAGTCTGGTCAAAACCCTCGTGAGGCTCAAAGATCGGCCGAGGCAC
gi|110341779|gb|DQ840196.1|  ATGCCAGAGGATGAGGTCTCGCGAGAGTCTGGTCAAAACCCTCGTGAGGCTCAAAGATCGGCCGAGGCAC
gi|431837877|gb|JX898859.1|  ATGCCAGAGGATGAGGTCTCGCGAGAGTCTGGTCAAAACCCTCGTGAGGCTCAAAGATCGGCCGAGGCAC
gi|431837883|gb|JX898862.1|  ATGCCAGAGGATGAGGTCTCGCGAGAGTCTGGTCAAAACCCTCGTGAGGCTCAAAGATCGGCCGAGGCAC
gi|431837857|gb|JX898849.1|  ATGCCAGAGGATGAGGTCTCGCGAGAGTCTGGTCAAAACCCTCGTGAGGCTCAAAGATCGGCCGAGGCAC
gi|431837853|gb|JX898847.1|  ATGCCAGAGGATGAGGTCTCGCGAGAGTCTGGTCAAAACCCTCGTGAGGCTCAAAGATCGGCCGAGGCAC
gi|431837873|gb|JX898857.1|  ATGCCAGAGGATGAGGTCTCGCGAGAGTCTGGTCAAAACCCTCGTGAGGCTCAAAGATCGGCCGAGGCAC
gi|431837865|gb|JX898853.1|  ATGCCAGAGGATGAGGTCTCGCGAGAGTCTGGTCAAAACCCTCGTGAGGCTCAAAGATCGGCCGAGGCAC
gi|431837861|gb|JX898851.1|  ATGCCAGAGGATGAGGTCTCGCGAGAGTCTGGTCAAAACCCTCGTGAGGCTCAAAGATCGGCCGAGGCAC
gi|431837867|gb|JX898854.1|  ATGCCAGAGGATGAGGTCTCGCGAGAGTCTGGTCAAAACCCTCGTGAGGCTCAAAGATCGGCCGAGGCAC
gi|431837863|gb|JX898852.1|  ATGCCAGAGGATGAGGTCTCGCGAGAGTCTGGTCAAAACCCTCGTGAGGCTCAAAGATCGGCCGAGGCAC
gi|431837859|gb|JX898850.1|  ATGCCAGAGGATGAGGTCTCGCGAGAGTCTGGTCAAAACCCTCGTGAGGCTCAAAGATCGGCCGAGGCAC
gi|431837855|gb|JX898848.1|  ATGCCAGAGGATGAGGTCTCGCGAGAGTCTGGTCAAAACCCTCGTGAGGCTCAAAGATCGGCCGAGGCAC
gi|431837869|gb|JX898855.1|  ATGCCAGAGGATGAGGTCTCGCGAGAGTCTGGTCAAAACCCTCGTGAGGCTCAAAGATCGGCCGAGGCAC
**PPR5                       ATGCCAGAGGATGAGGTCTCGCGAGAGTCTGGTCAAAACCCTCGTGAGGCTCAAAGATCGGCCGAGGCAC
**PPR4                       ATGCCAGAGGATGAGGTCTCGCGAGAGTCTGGTCAAAACCCTCGTGAGGCTCAAAGATCGGCCGAGGCAC
gi|431837871|gb|JX898856.1|  ATGCCAGAGGATGAGGTCTCGCGAGAGTCTGGTCAAAACCCTCGTGAGGCTCAAAGATCGGCCGAGGCAC
**PPR3                       ATGCCAGAGGATGAGGTCTCGCGAGAGTCTGGTCAAAACCCTCGTGAGGCTCAAAGATCGGCCGAGGCAC
gi|431837875|gb|JX898858.1|  ATGCCAGAGGATGAGGTCTCGCGAGAGTCTGGTCAAAACCCTCGTGAGGCTCAAAGATCGGCCGAGGCAC
**PPR1                       ATGCCAGAGGATGAGGTCTCGCGAGAGTCTGGTCAAAACCCTCGTGAGGCTCAAAGATCGGCCGAGGCAC
gi|431837881|gb|JX898861.1|  ATGCCAGAAGATGAGGTCTCGCGAGAGTCTGGTCAAAACCCTCGTGAGGCTCAAAGATCGGCTGAGGCAC
gi|431837879|gb|JX898860.1|  ATGCCAGAAGATGAGGTCTCGCGAGAGTCTGGTCAAAACCCTCGTGAGGCTCAAAGATCGGCTGAGGCAC
**PPR2                       ATGCCAGAGGATGAGGTCTCGCGAGAGTCTGGTCAAAACCCTCGTGAGGCTCAAAGATCGGCCGAGGCAC
gi|110341785|gb|DQ840199.1|  ATGCCAGAGGATGAGGCCTCGAGAGAATCCGGCCAAACCCCTCGTGAGGCTCAGAGGTCGGCTGAGGCAC

                                       290       300
                             ....|....|....|....|....
gi|110341787|gb|DQ837640.1|  TCTTCAGGCTGCAGGCCATGGCCA
gi|110341775|gb|DQ840194.1|  TCTTCAGGCTGCAGGCCATGGCCA
gi|307646466|gb|HQ131938.1|  TCTTCAGGCTGCAGGCCATGGCCA
gi|307646464|gb|HQ131937.1|  TCTTCAGGCTGCAGGCCATGGCCA
gi|110341777|gb|DQ840195.1|  TCTTCAGGCTGCAGGCCATGGCCA
gi|110341761|gb|DQ840187.1|  TCTTCAGGCTGCAGGCCATGGCCA
gi|110341757|gb|DQ840185.1|  TCTTCAGGCTGCAGGCCATGGCCA
```

gi|110341783|gb|DQ840198.1|	TCTTCAGGCTGCAGGCCATGGCCA
gi|110341781|gb|DQ840197.1|	TCTTCAGGCTGCAGGCCATGGCCA
gi|110341779|gb|DQ840196.1|	TCTTCAGGCTGCAGGCCATGGCCA
gi|431837877|gb|JX898859.1|	TCTTCAGGCTGCAGGCCATGGCCA
gi|431837883|gb|JX898862.1|	TCTTCAGGCTGCAGGCCATGGCCA
gi|431837857|gb|JX898849.1|	TCTTCAGGCTGCAGGCCATGGCCA
gi|431837853|gb|JX898847.1|	TCTTCAGGCTGCAGGCCATGGCCA
gi|431837873|gb|JX898857.1|	TCTTCAGGCTGCAGGCCATGGCCA
gi|431837865|gb|JX898853.1|	TCTTCAGGCTGCAGGCCATGGCCA
gi|431837861|gb|JX898851.1|	TCTTCAGGCTGCAGGCCATGGCCA
gi|431837867|gb|JX898854.1|	TCTTCAGGCTGCAGGCCATGGCCA
gi|431837863|gb|JX898852.1|	TCTTCAGGCTGCAGGCCATGGCCA
gi|431837859|gb|JX898850.1|	TCTTCAGGCTGCAGGCCATGGCCA
gi|431837855|gb|JX898848.1|	TCTTCAGGCTGCAGGCCATGGCCA
gi|431837869|gb|JX898855.1|	TCTTCAGGCTGCAGGCCATGGCCA
**PPR5	TCTTCAGGCTGCAGGCCATGGCCA
**PPR4	TCTTCAGGCTGCAGGCCATGGCCA
gi|431837871|gb|JX898856.1|	TCTTCAGGCTGCAGGCCATGGCCA
**PPR3	TCTTCAGGCTGCAGGCCATGGCCA
gi|431837875|gb|JX898858.1|	TCTTCAGGCTGCAGGCCATGGCCA
**PPR1	TCTTCAGGCTGCAGGCCATGGCCA
gi|431837881|gb|JX898861.1|	TCTTCAGGCTGCAGGCCATGGCCA
gi|431837879|gb|JX898860.1|	TCTTCAGGCTGCAGGCCATGGCCA
**PPR2	TCTTCAGGCTGCAGGCCATGGCCA
gi|110341785|gb|DQ840199.1|	TCTTCAGGCTGCAAGCTATGGCCA
